# Control of Coherent Light through Microperiodic Director Modulation in Nematic Films under Low-Voltage DC Electric Field

**DOI:** 10.3390/ma16176014

**Published:** 2023-09-01

**Authors:** Georgi B. Hadjichristov

**Affiliations:** Laboratory of Optics and Spectroscopy, Georgi Nadjakov Institute of Solid State Physics, Bulgarian Academy of Sciences, 72 Tzarigradsko Chaussee Blvd., 1784 Sofia, Bulgaria; georgibh@issp.bas.bg

**Keywords:** nematic liquid crystals, optical phase grating, coherent optical processes, light scattering, light diffraction, laser beam intensity control

## Abstract

This work addresses the achievement of efficient control of laser light transmission through stationary microperiodic parallel stripe textures formed in films of nematic liquid crystals (NLCs) in planar-oriented cells upon a direct-current (DC) electric field. By varying the field intensity and, thereby, the field-induced periodic modulation of the nematic director and hence the complex transmittance function corresponding to the longitudinal domain texture induced in NLC films with initial planar alignment, the intensity of a linearly polarized laser beam passed through the films can be well controlled. In 25 µm-thick films of room-temperature NLCs pentylcyanobiphenyl (5CB), this results in a low-voltage (~4 V) sharp and deep V-shaped behavior of their electro-optically controlled transmittance. Such a reversible electro-optical effect is interesting for active control of laser beam intensity and other applications. The relevant physical mechanism is analyzed and explained.

## 1. Introduction

Among the variety of liquid crystal (LC) textural formations [1,2,3], field-induced spatial patterns and ordered textures offer attractive possibilities for field-commanded effects and applications, such as controllable shifting, angular deflection, scattering, and diffraction of light, for use in diffractive, adaptive, and non-linear optics, along with microscopy and electro-optics [4,5]. In some cases, regular field-induced grid-like patterns based on periodic modulations and orientation patterns in LC media, in particular nematic LCs (NLCs), are suitable to use in optical devices, such as optical switches and filters for laser beams, optical phase gratings, similarly to the well-known various types of electrically-driven diffraction gratings in NLCs, e.g., [6], and electro-optically addressed NLC tunable diffraction and phase gratings, e.g., [7,8]. Such spatial, polarization, and phase modulators of light have found useful applications in photonics, optical information processing and fiber-optic communications [9,10,11], laser beam steering [12,13], programmable shaping of femtosecond laser pulses [14,15], reconfigurable generation of optical vortices for manipulation of laser beams and light pattern formation [16,17] and in other modern scientific research fields.

Nowadays, NLC gratings with generated spatial patterns and thus customized diffraction patterns have attracted much attention in both industry and scientific research due to their simple preparation, cost effectiveness, and high performance, including diffraction efficiency, tuneability, and polarization sensitivity [18,19,20,21]. Diffraction grating effects exhibited by homogeneously aligned NLC layers with a microperiodic distortion of their director field were thoroughly analyzed and elucidated [22,23,24]. The appearance and characteristics of electric field-driven texture patterns in NLC layers depend on both dielectric permittivity anisotropy and electrical conductivity anisotropy, the initial director orientation, as well as other initial conditions, system parameters, the LC cell, the characteristics of the NLC material, and possibly the additives included in it. Relevant physical mechanisms (electrostatic, electrohydrodynamic, and other standard and nonstandard models, including flexoelectricity) have been developed to describe and explain various field-induced textural formations in NLCs [3,25,26,27,28,29,30,31,32].

Of special research and practical interest are some types of electric field-induced spatially periodic and highly regular and stable stripe patterns formed in NLC materials. Such longitudinal domains (LDs) in NLC films have been comprehensively investigated both theoretically and experimentally by electrical, dielectric, and electro-optical (EO) measurements [33,34,35,36,37], including numerous diffraction studies in planarly aligned NLC layers, e.g., [22,24,36,38,39,40].

In the work presented here, scientific and applied physics interest is focused on LDs formed in planarly-aligned films of NLCs, for instance, pentylcyanobiphenyl (5CB), exposed to a direct current (DC) electric field. 5CB has a stable nematic phase at room temperature and a relatively high positive dielectric anisotropy, and planar cells with this NLC exhibit well-developed electric field-induced LDs [37]. The present experimental study of DC voltage-controlled laser beam transmission, diffraction, and scattering in planar 5CB nematic films demonstrates effective and low-voltage EO control of the intensity of the laser beam passed through them utilizing DC voltage-induced microperiodic director-modulation textures. Extending previous works [41,42], a more detailed investigation of DC voltage-commanded laser beam transmission, diffraction, and scattering by planar cells with 5CB was correlated with optical microscopy observations and with results obtained from polarization and time-domain measurements. The effect observed was also characterized with respect to the laser beam wavelength and the angular orientation of the 5CB films relative to the incident laser beam. The advantages, limitations, and applicability of the proposed approach for enhancing the optical contrast ratio of coherent light transmission through nematic films were discussed. The aim of this investigation is not to explore in detail the microperiodic director modulations in NLC layers themselves, their physics, and the various characteristics of the electrically-induced spatial patterns (domains) in the studied NLC materials, but to investigate and identify the effect of such well-known for a long time textures on the transmission of coherent light through NLCs films and to put them into action for practical use through the EO effect observed. The textural domains in the studied planar NLC films were characterized only to the extent necessary for the EO application proposed here.

## 2. Materials and Methods

### 2.1. NLCs Films

Experiments were carried out on planarly-aligned samples of the NLCs 4′-*n*-pentyl-4-cyanobiphenyl (5CB) and 4′-*n*-heptyl-4-cyanobiphenyl (7CB) supplied by Merck and used as received. These NLCs are members of the cyanobiphenyl family, which was one of the first commercially available nematic materials for use in LC displays. Due to their outstanding properties at room temperature, i.e., high chemical stability and sensitivity to applied electric fields, they are still commonly employed. At room temperature, both compounds exhibit a stable nematic LC phase. They are optically birefringent materials. For instance, 5CB is characterized by extraordinary and ordinary refractive indices *n_e_* = 1.706 and *n*_0_ = 1.532, respectively, at wavelength λ = 633 nm and 25 °C [43]. 5CB and 7CB have a relatively large positive dielectric anisotropy, Δε = ε_||_ − ε_⊥_ = 8.2 and Δε = 6.7, respectively, at 20 °C and at an electric field frequency *f* = 1 kHz [44]. The value of the real part of the dielectric susceptibility of these nematics along the preferred molecular direction (ε_||_) is about twice higher than that in the transverse direction (ε_⊥_) [44].

Planar films of NLCs with a planar alignment were prepared in commercial flat-panel LC glass cells (KSRO-25/B111N1NSS Up/Low, manufactured by E.H.C. Co. Ltd., Tokyo, Japan) with a 25 µm gap. The inner surfaces of the two glass plates of the cells were covered (by the manufacturer) with an ultrathin, transparent, electrically conductive layer of indium-tin-oxide (ITO) that served as electrodes. The ITO glasses had a polyimide overcoat with unidirectional rubbing. In 5CB films in such LC cells, stable DC-induced texture formations, such as those studied here, are absolutely reliably induced.

The NLCs in the isotropic liquid phase were injected into the cells by capillary forces. Before injecting, the cells and the NLCs were heated to a temperature above the clearing point (the nematic-isotropic phase transition temperature) in order to avoid a non-uniform alignment of the NLCs. The formation of the nematic LC phase was established with polarizing optical microscopy (POM) by observing birefringence between crossed polarizers. The temperature range for the nematic phase of 5CB was 24–35 °C and 28.5–42 °C for 7CB. The parallel-rubbed polyimide ultrathin surface layers provide NLC molecular alignment—they force the confined NLC molecules to orient themselves homogeneously parallel to the rubbing direction. The quality of the orientation of the nematic films was checked using POM. The strong planar alignment of NLCs with an overall orientation of the nematic director along the rubbing direction of the cells was confirmed.

### 2.2. Electro-Optical Measurements

The optical transmittance and diffraction from the prepared nematic films were investigated using a non-focused beam of He-Ne laser HNL050RB (Thorlabs GmbH, Munich, Germany) operating at a wavelength (λ) of 632.8 nm and having an optical power of 5 mW and optical noise of less than 0.2%. The linear polarization of the laser beam (>10^4^:1) was selected by the rotatable Thorlabs LPVISB050-MP nanoparticle linear film polarizer. With this polarizer in Thorlabs’ KS05RS kinematic rotation mount, one can set the polarization direction of the laser beam with an accuracy of ±0.5°.

Some tests were performed with a temperature-stabilized diode-pumped continuous wave (c.w.) solid-state laser DPGL-4007 (Photop Suwtech Inc., Shanghai, China), with Nd:YVO4 crystal, λ = 532 nm, 100 mW, linearly polarized (>500:1)). Produced by intracavity second harmonic generation, the output of this laser source did not contain IR radiation. Both green and He-Ne laser beams had TEM_00_ spatial profiles with a Gaussian intensity distribution and a divergence of 0.8 mrad and 1 mrad, respectively. The power stability of these laser sources was better than ±0.5%. A 5 mW laser diode emitting at 405 nm (beam divergence ~1.5 mrad) was also used.

The LC cells were mounted on a micro-manipulating translation-rotation stage. This allows illumination at a desired angle of incidence of the laser beam. In most of the experiments, the incident laser beam was directed normally to the nematic film plane (or, more correctly, nearly normally, to avoid optical interference from reflections from optical elements). A part of the film about 2 mm in diameter was illuminated. The laser power incident on the nematic films was kept at ~1 mW.

**Figure 1:**
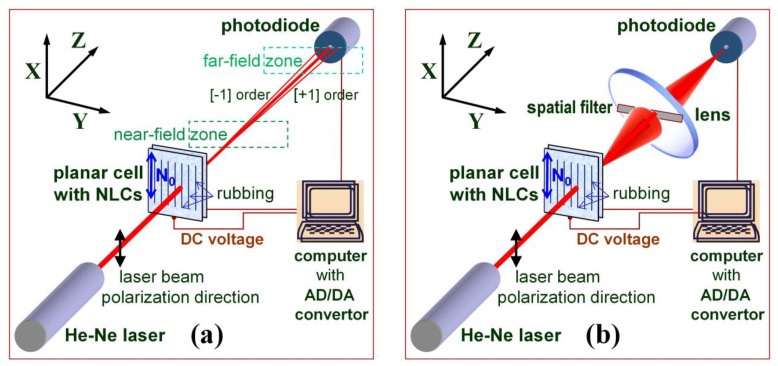
Schematic of the experimental setup for measurement of coherent light transmission and diffraction (**a**) and coherent light scattering (**b**) of a laser beam behind the cell with NLC film in the experiments in this work.

A DC electric field was applied across the two ITO-coated glass plates of the LC cell (i.e., the electric field direction coincided with the laser beam direction). The electrically active area of the cells was 10 mm × 10 mm. The experiment setup for EO measurements is shown in Figure 1. The light transmitted through the 5CB cells was detected by a photodiode. For measurement of the spatially selected forward spread of scattered light, a large-aperture lens assembly was used to collect and focus the light onto a photodiode (as schematically shown in Figure 1b). In this case, proper spatial filtering of the light was performed. As for the light of diffraction peaks and other fine localized diffraction features, they were carefully separated by a small circular aperture (iris diaphragm properly open) or by a pinhole (1 mm diameter) in front of the photodiode (Figure 1a). When the laser beam incidence angle was varied, a large-aperture photodiode was used in the measurements. The *X* and *Y* coordinate axes in the *XYZ* reference system shown in Figure 1 are related to the film plane, with the *X* axis parallel to the initial orientation of the nematic director (denoted as **N_0_**, at zero field).

The light intensity was measured using a multi-channel digital-analog/analog-digital conversion interface card (Decision Group Inc., Taiwan, China) installed in a computer slot for programmable data acquisition. This high-precision data conversion card provides both the driving DC voltage and the digitization of the photodiode signal in a range of 12 bits with a conversion time of 60 µs. The computer-generated digital-to-analog pulse formation by the card is characterized by a current setting time of 0.5 µs and nonlinearity of less than 0.2%. The behaviors of DC voltage-dependent laser beam transmittance and diffraction/scattering behind the cells were recorded in voltage steps of 0.1 V. The interval between the data acquisitions was equal to 30 s, and the averaging of 10 measurements was done during 10 s at each step. The pause of 30 s was sufficient to attain the stationary state of the nematic in the cells.

In the series of measurements in which the angle of the polarization direction of the incoming laser beam was varied, the light intensity measurements were carried out under identical experimental conditions and using a reference channel to control the laser beam intensity in order to accurately determine the intensity of the detected light transmitted through the cell. In the experiments in which the EO effect with a high dynamic range was measured, a low-noise photodetector with an optical-power working range of 1–10^6^ and a measurement uncertainty of ±3% was employed—a Thorlabs PM100 power-meter equipped with a silicon photodiode power-sensor S120VC. The lower limit of this unit was 10–50 nW (in the dark).

The temperature of the studied cells was maintained by a Mettler FP82 hot stage and was controlled with an accuracy of 0.1 °C. In most experiments, the temperature was stabilized at 25 °C. Also, measurements by varying the temperature of the cells in the range of 25–32 °C were performed. A polarizing optical microscope NU-2 (Carl Zeiss Jena GmbH, Jena, Germany) was used to observe pattern formation in the studied LC cells. DC electric field-induced texture changes in the prepared NLC films were inspected in the voltage range of 0–10 V. Images of coherent light diffraction/scattering resulting from a He-Ne laser beam passed through the NLC cells were displayed on an imaging screen placed transversally to the beam behind the cells. Pictures and videos of the diffraction pattern were taken with a digital camera VG-130-D-715, 4300 × 3200 pixels (Olympus Imaging China Co. Ltd., Beijing, China) in the dark (with room lights off). The images of light patterns were processed and analyzed with Linux IMAL software (version 6.0).

## 3. Results and Discussion

### 3.1. Longitudinal Domain Texture in 5CB Planar Cells under DC Electric Field: Polarizing Optical Microscopy Data

At an appropriate DC voltage (*V_DC_*) applied to the prepared 5CB films, their morphology displays regular parallel-striped textures well observable by optical microscopy. Two types of such textural formations were distinguished: wide-formed longitudinal domains and narrow-striped rubbing-induced longitudinal texture.

#### 3.1.1. Wide-Formed Electrically-Induced Longitudinal Domains

**Figure 2:**
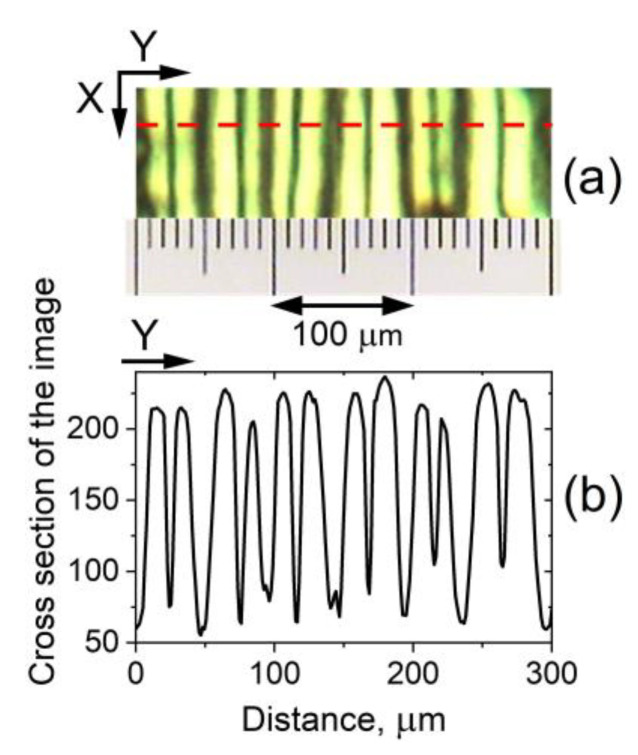
(**a**) POM image of the texture formed in planar 5CB film (thickness 25 μm) at *V_DC_* = 4.5 V. The micrograph was taken by a slightly uncrossed polarizer and analyzer with their axes in the *X* and *Y* directions, respectively (see Figure 1). The polarization of the input light was parallel to the initial alignment of the NLCs (the rubbing of the cell plates along the *X* direction). (**b**) Cross-sectional profile of texture image (**a**) digitized at the section along a preselected line indicated by a red dashed line.

When the voltage *V_DC_* applied to the initially planarly oriented 5CB films was above a well-defined threshold value of *V_form_* = 4.2 V, stationary stripe texture patterns in these films were clearly visible by POM (Figure 2a). The patterns were parallel to the direction of the rubbing of the cell plates—the initial orientation of the 5CB LC molecules (the direction *X*, see Figure 1). Such DC field-induced longitudinal texture formations in nematic planar cells (along the planar director orientation at the confining slides of the cells) are well known, e.g., LDs reported by Aquire and co-workers in their comprehensive study of regular structures in 5CB NLCs under the joint action of DC and alternating-current (AC) voltages [37].

In our case, the stationary LDs can be attributed to flexoelectric domains. Similar domains are long known for NLC films under DC, very low-frequency AC, or the joint action of DC and AC electric fields. Such textures depend very strongly on the conditions (the applied voltage, the NLC film thickness, the state of the boundaries, their treatment, the molecular anchoring at the walls, and other factors) [1,45,46,47,48,49,50]. The physical mechanisms of the domain’s appearance have been studied for years by numerous research groups. The observed bright LDs are divided by thin dark stripes in the middle (Figure 2a). The width of the LDs was larger than the cell thickness. For the texture shown in Figure 2a, a mean width of 46.7 µm (standard deviation ± 4.5 µm) was estimated by averaging over a lot of cross-sectional profiles of the obtained micrographs. At different locations on the film, the length and number of the LDs vary, suggesting a surface contribution to domain formation.

The stationary periodic LDs result from the static deformations of the nematic director, whose initial orientation **N_0_** (in the absence of an applied electric field) is in the direction *X* (see Figure 1). As a sequence of alternating dark and bright stripes observable by POM, these domain patterns represent a periodic spatial modulation of the director. Using POM, their best contrast was achieved when the polarizer and analyzer were a few degrees off the perpendicular, suggesting that the periodic modulations of the director are exclusively in the plane of the films (the *X*–*Y* plane). Additional observations indicated that the spatial patterns shown in Figure 2a are flexo-dielectric walls (more specifically, dielectric flexoelectric walls) [1,51]. This static flexoelectric instability is different from the flexoelectric domains of the Vistin-Pikin-Bobylev type [45,46,47,48,49]. The latter type of patterned flexoelectric instability is a bulk flexoelectric effect occurring in nematic layers with relatively strong planar anchoring at the substrates. Such domains formed along the initial orientation of the nematic are volume flexoelectric deformations. In contrast, the flexo-dielectric walls present flexoelectric deformations in the bulk of the planar nematic film but very near the electrodes of the LC cell.

**Figure 3:**
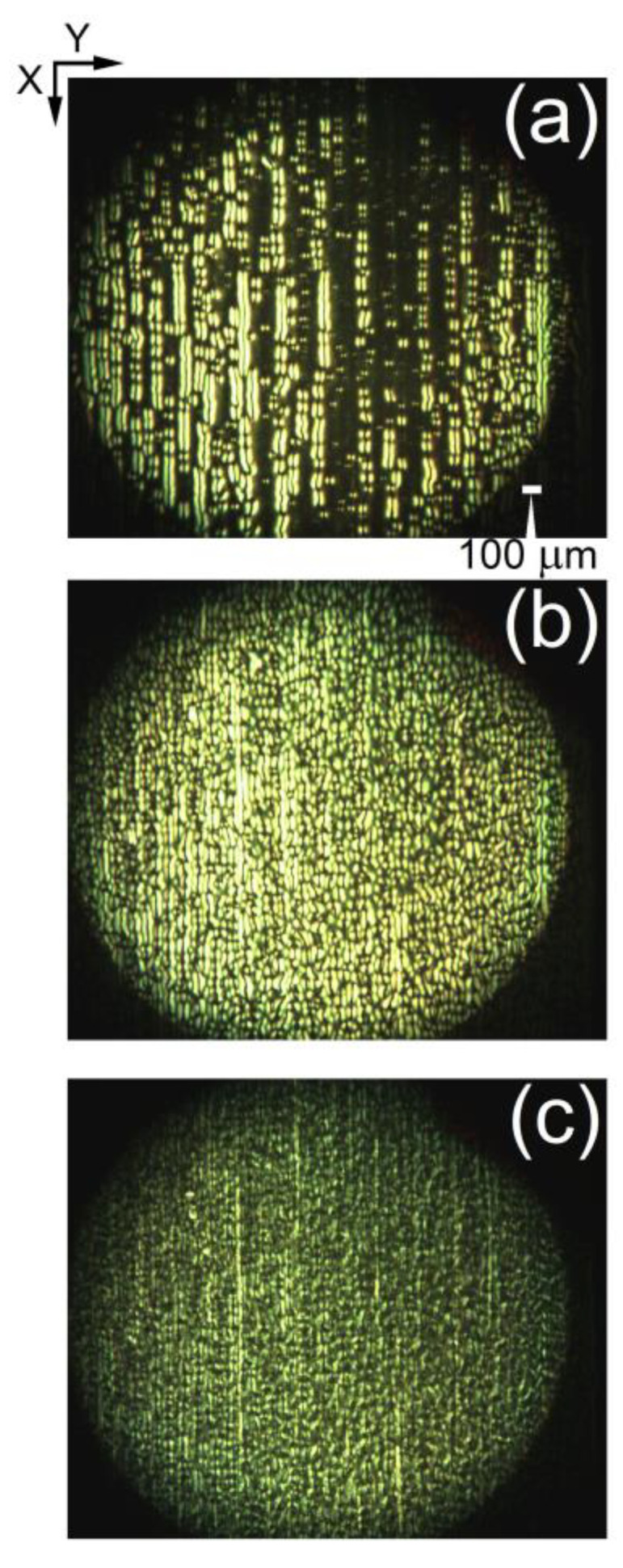
As in Figure 2a, photomicrographs captured for 5CB nematic film in planar cell at DC voltage: 4.5 V (**a**); 5.8 V (**b**) and 6.5 V (**c**).

Some inhomogeneity in the LD texture pattern can be present over the 5CB films. There were regions with less or more regular formation of LDs (Figure 3). This arises from the boundary conditions at the confining plates of the LC cell and from the inhomogeneity of the anchoring of the NLC molecules. By POM, the LDs were also visible at *V_DC_* > *V_form_* (Figure 3a), but a stable periodic array of parallel stripes was observed in a relatively short voltage range. At *V_DC_* higher than 5.8–6 V, undulations and fragmentation of the LDs started to develop (Figure 3b). At *V_DC_* > *V_hd_* = 6.5 V the electrically induced hydrodynamic processes [52] in the 5CB cells were enhanced to a degree that disrupted the LDs, clearly visible under the microscope (Figure 3c). It should be noted that the voltage value *V_hd_* varies slightly; e.g., the same 5CB in other but identical planar cells also shows *V_hd_* of about 6 V or slightly less. On the other hand, for some of the prepared 5CB films, this periodic texture could retain up to 8 V. With decreasing *V_DC_* in the same range, from 10 V to 0 V, the morphological changes were repeated in reverse at the corresponding voltage values (after the short time, less than 30 s, necessary for the stationary state of the NLC soft material in the cells, monitored by an oscilloscope during the experiments). Also, it was checked that the EO V-shaped curve of the 5CB film is fully repeatable after heating the LC cell above the temperature of the NLC-to-isotropic phase transition of 5CB (ca. 34 °C) and by subsequent cooling to room temperature. In doing so, the nematic phase and EO properties of the 5CB nematic were recovered, and the voltage values *V_th_* and *V_hd_* were the same as before heating.

#### 3.1.2. Narrow-Striped Rubbing-Induced Longitudinal Texture

**Figure 4:**
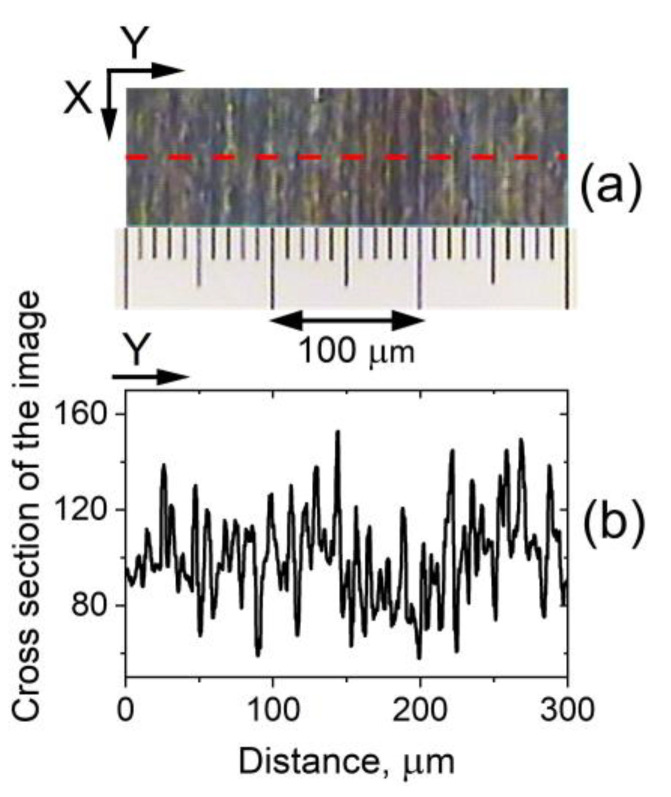
(**a**) POM image of the texture formed in a 25 μm-thick planar-oriented layer of nematic 5CB at *V_DC_* = 3.3 V. Slightly uncrossed polarizers (set along *X* and *Y*). The input light polarization was parallel to the initial alignment of the NLCs (i.e., along *X*—in the direction of the rubbing of the cell plates); (**b**) Cross-sectional profile of texture image (**a**) digitized at the section along a preselected line indicated by a red dashed line.

POM also revealed the presence of a closely spaced, narrow-formed LD texture in the 5CB planar cells under study. As with the aforementioned type of wide-formed *V_DC_*-induced LDs, this microtexture was also along the *X*-axis and was stable over time. These spatial patterns are due to the static microperiodic orientational modulation of the director and appear as fine quasi-periodic parallel stripes along the rubbing of the LC cells. The so-formed quasi-linear grating of deformations (pattern consisting of alternating dark and bright stripes, Figure 4a) was most easily viewed by shadowgraph technique or by POM between slightly uncrossed polarizers when the applied voltage is below *V_form_*. The narrow-striped director modulation was also present at the zero field. The observed fine-stripe texture follows the regular scratches –the rubbing of the cell plates, which determine the formation of the initial NLC texture. The spatial periodicity of the stripes along *Y* is estimated to be 6–8 μm (Figure 4b).

POM studies show that EO changes in the microscopic optical response of these textural formations under an applied DC voltage in the range of 1–10 V were relatively slow and hardly observable. The narrow-striped LDs are surface irregularities called by Hinov et al. “rubbing-induced domains”, or more precisely “rubbing-induced surface texture” [53]. They are due to complex flexoelectric effects and are located in a very thin region with a thickness of ~1 μm close to the electrodes of the LC cell [53]. At *V_DC_* > *V_form_*, these fine stripes were “suppressed” by the wide-formed field-induced LDs discussed in the previous Section 3.1.1. The DC field-induced flexoelectric changes of these textural formations are related not only to bulk interactions but also to surface interactions that depend on the planar anchoring of the LC molecules at the substrates.

### 3.2. Interaction of Coherent Light with LDs in 5CB Planar Films under DC Electric Field

As molecular orientation patterns, both types of DC electric field-induced spatially periodic parallel textures in planar nematic film (Section 3.1) can be considered diffraction gratings (in spectral regions where the nematic material is transparent, or at least not strongly absorbing). As such, when coherent light interacts with a planar nematic film, the LDs can give rise to characteristic diffraction patterns.

#### 3.2.1. Optical Diffraction by Wide-Formed Electrically-Induced LDs

From an application point of view, the electrically formed spatially periodic array of LDs in 5CB planar nematic films is attractive for producing optical diffraction. For textures shown in Figure 2 and Figure 4, the Klein–Cook parameter [54], defined as *Q* = 2 π λ_0_ *L*/Λ^2^ *n*_0_ (where λ_0_ is the wavelength of light in vacuum, *L* is the interaction length (active grating thickness), Λ is the grating spacing, and *n*_0_ is the mean refractive index), is *Q* << 1, i.e., the interaction of the laser beam with the LDs is under Raman–Nath conditions (at normal incidence of light). Since the diameter (*D* = 2 mm) of the spot of the incident laser beam (a plane wave) on the surface of the studied NLC film is much greater than, Λ, the Raman–Nath diffraction regime predicts that the diffracted light in the far-field zone consists of sharp and well-separated lines—the diffraction pattern contains many diffraction orders with intensities given by Bessel functions (not taking into account propagation diffraction effects inside the NLC film, which give rise to a far-field diffraction less or more smeared out).

**Figure 5:**
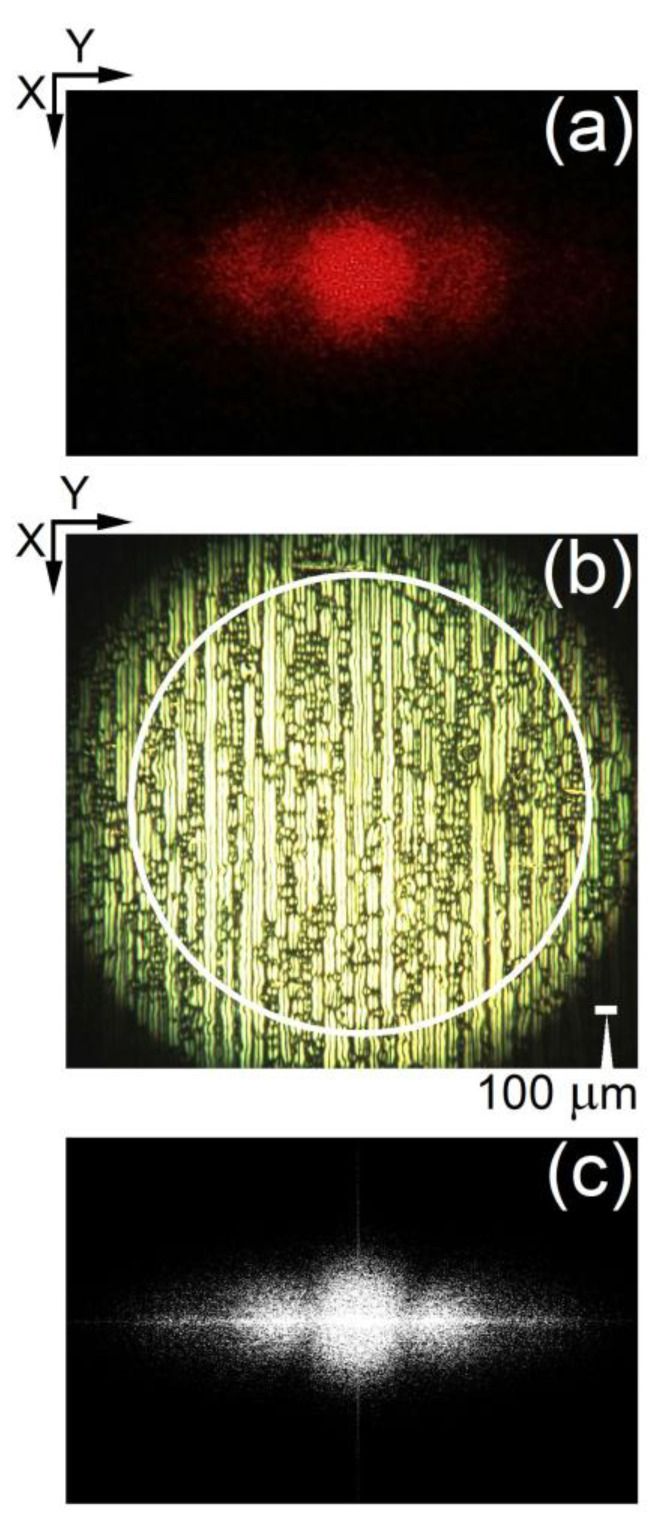
(**a**) Diffraction pattern obtained with a laser beam (He-Ne laser, λ = 632.8 nm) incident on a planar 5CB film (thickness 25 μm) at *V_DC_* = 5.2 V. The diffraction was registered in transmission, in the near-field zone; the cell-to-screen distance was 15 cm. The polarization of the incident laser beam was along the direction of the rubbing of the 5CB cell; (**b**) Optical microscopy image of the texture formed in the 5CB film at *V_DC_* = 5.2 V. A low magnification (×4) of the microscope was used in order to scale the image as large as the spot diameter of the incoming laser beam (indicated with a circle); (**c**) Fourier transform of image (**b**).

Upon illumination with a He-Ne laser beam whose polarization is along the LDs (i.e., the direction of the rubbing of the cell plates, the *X* axis, Figure 1a) and when the voltage applied to the 5CB planar cell is above *V_form_* (Figure 5a), two bright lateral diffraction peaks arise from these periodic textural formations. The observed diffraction pattern seems to be Fraunhofer diffraction, such as those from a thin harmonic diffraction grating (see, for example, [55]). No diffraction was present when the beam polarization direction was orthogonal to the rubbing of the cell plates. The diffracted light intensity was predominantly distributed in two side maxima (the first-order diffraction peaks, numbers +1 and −1, respectively) in addition to the central peak associated with the zero-th-order diffracted light. Behind the 5CB cell, this triplet light beam was spatially localized and had a relatively low spatial divergence (Figure 1a). The divergence of the zero-th-order diffracted light beam was a little higher than that of the incident laser beam. 

Fourier transform (Figure 5c) of a typical texture image of the studied 5CB cells with well-developed LDs, i.e., at *V_DC_* well above *V_form_* (e.g., the image shown in Figure 5b), is close to the diffraction pattern (Figure 5a) observed when the same LD texture (keeping the corresponding value of *V_DC_*) was illumined with highly coherent light (monochromatic and low-divergent). This suggests that the regular field-induced LD texture in the 5CB cells acts as an electrically driven diffraction grating when illuminated by a monochromatic plane light wave.

**Figure 6:**
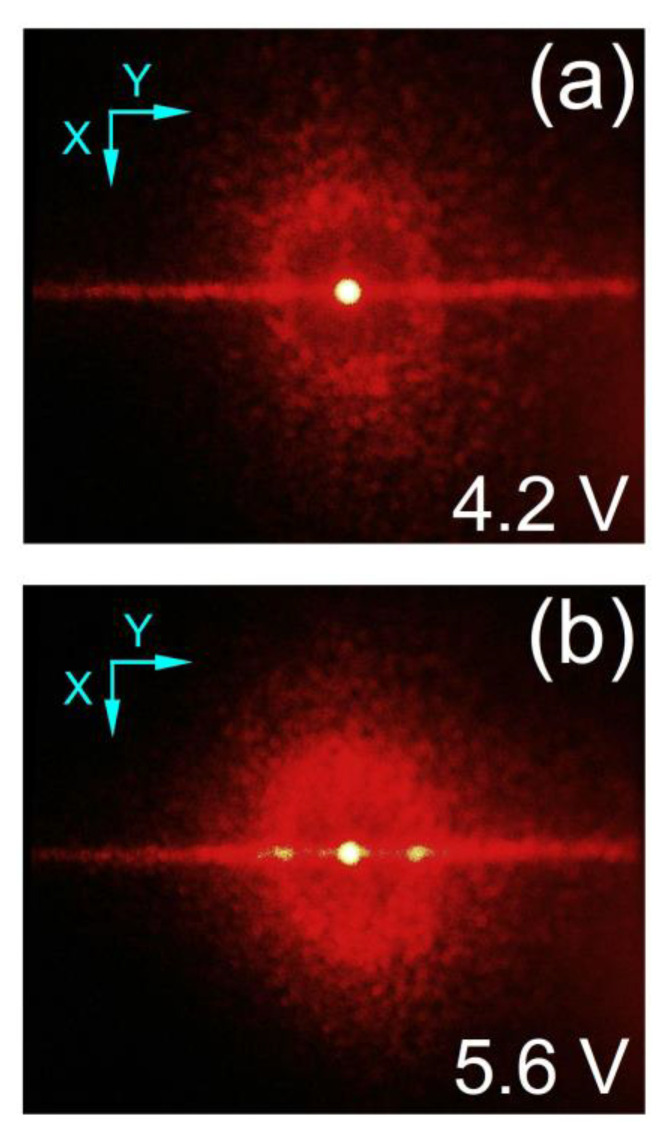
Far-field diffraction pattern observed on a white-paper transversal screen behind a planar cell with 5CB film with a thickness of 25 μm. The cell was illuminated with a He-Ne laser beam whose polarization was along the direction of the rubbing of the cell plates (direction *X*). The pictures were taken under identical conditions; cell-to-screen distance of 85 cm. The DC voltage applied to the cell: *V_DC_* = 4.2 V (**a**) and 5.6 V (**b**).

Figure 6 shows far-field diffraction patterns from a He-Ne laser beam propagating through a 5CB planar cell at *V_DC_* > *V_form_*. The spatial frequency of the formed LD grating texture (the grating period), calculated from the measured angular spacing of the features in the triplet diffraction pattern in Figure 6b, was Λ~45 μm, in accordance with the average width of LDs, estimated from optical micrographs (see Section 3.1.1). Note that the spatial period of the resulting texture pattern (Figure 2b) estimated by the cross-sectional profiles of the micrographs was 23–25 µm (depending on the examined location on the 5CB layer).

#### 3.2.2. Coherent Light Scattering/Diffraction by Striped Textures in 5CB Planar Films under DC Electric Field

##### Diffraction Pattern

**Figure 7:**
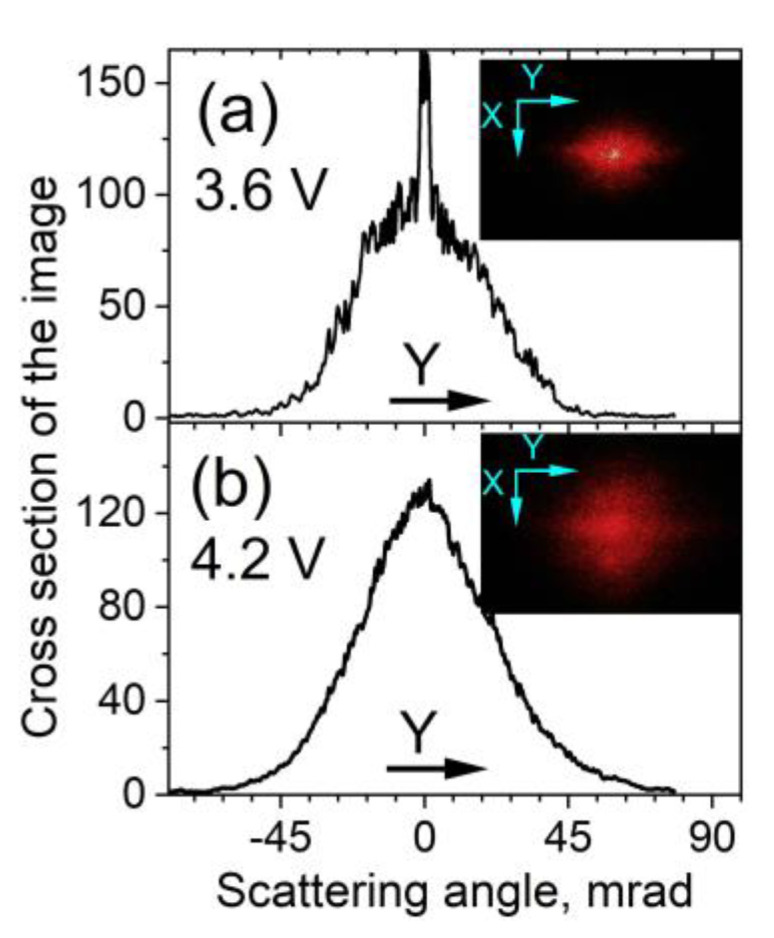
Near-field pattern of forward CLS behind planar cell with 5CB film (25 μm thickness) upon DC electric field: *V_DC_* = 3.6 V (**a**) and *V_DC_* = 4.2 V (**b**). The images were captured on a black-paper transverse screen, screen-to-cell distance of 17 cm. The polarization of the incident He-Ne laser beam was parallel to the rubbing of the cell plates (the direction *X*). The corresponding digitized horizontal/equatorial cross-sectional profiles of CLS intensity spatial distribution (angular spread of the light intensity) are plotted.

Besides Fraunhofer diffraction, another coherent optical process, namely coherent light scattering (CLS), was also present for planarly aligned 5CB films under a DC electric field. This process results in a distinctly localized spatial pattern of diffuse scattered light, seen as a halo or ring in Figure 6. As experimentally observed for the studied 5CB films, CLS arises at DC voltages lower than *V_form_* (see Section 3.1.1), i.e., at increasing *V_DC_*, CLS precedes the Fraunhofer diffraction from the wide LDs discussed in Section 3.2.1. Well visible in the near-field zone behind the 5CB cells, a strong CLS of the laser beam propagating through the 5CB planar film appears as a diffuse diffractive halo of speckle light (speckle diffraction patterns being random fluctuations in the multiply scattered intensity of coherent light). The light from CLS registered on a transversal screen as a halo (Figure 7) and was spatially spread in a light cone around the direction of the propagating laser beam (Figure 1b). The cone angle of diffuse CLS was roughly estimated to be at least 0.025 sr, as measured at a full-width-at-half-maximum intensity of the light behind the 5CB cell at *V_DC_* = 4.2 V (Figure 7b). It should be noted that such an angular spread is considerably larger than that of light scattering from spontaneous fluctuations of anisotropy in NLCs without an electric field applied, e.g., [56,57].

The relatively bright, narrow, widely spread horizontal/equatorial light pattern across the center of the overall scattering/diffraction pattern from the studied 5CB cells, seen more clearly in Figure 6, is also diffuse-like and can be considered CLS. This stripe light pattern extended along a direction perpendicular to the rubbing direction of the cell plates is similar to the effect of a fine quasi-linear diffraction grating, such as one with multiple vertical slits spaced very close together. In the present case, such multiplex diffraction should be the result of a large number of narrow (micrometer) stripes formed parallel to the rubbing direction of the examined cells with planar 5CB films. More precisely, the observed elongated diffuse light pattern should be called “diffuse-diffraction stripe spatial spread/spectrum” (hereafter called DDS), being complex overlaps of many diffraction orders. In the experiment, this diffraction feature, oriented orthogonally to the rubbing direction of the LC cell, can be used for very precise adjustment of the cell with respect to the polarization direction of the linearly polarized incident laser beam.

The observed CLS is due to field-induced static inhomogeneities of NLC director field distribution (in the bulk and on the cell walls). In our case, the refractive index gradients corresponding to the field-induced orientation pattern can be associated with the generation of an array of very thin cylindrical lenses [58,59]. The stationary field-induced narrow-striped LDs in the 5CB planar nematic films can be regarded as such an array for the extraordinary component of the light incident normally to the films. When the orthogonal size (the width) of the stripes of such a parallel orientation pattern is small and of the order of the wavelength of the light transmitted through the array of LDs, then light scattering from such an electrically induced spatially periodic array takes place. 

In general, CLS from the examined 5CB cells is due to all micro-sized irregularities and regularities in the illuminating volume and on the cell surfaces, including the microperiodic orientational modulation of the director appearing as fine quasi-periodic parallel stripes along the rubbing of the cells, discussed in Section 3.1.2. Produced by the interference of many diffraction fields, CLS from electrically-formed quasi-periodic micro-scale director spatial modulation can be regarded as diffraction from a multi-frequency grating associated with an LD periodicity that exhibits many spatial frequencies. In fact, many irregular stripes illuminated by the laser beam within the laser beam spot area on the surface of the LC cell correspond to many grating periods. The intensity distribution of the resulting light diffraction will be an overlap of many diffraction functions; hence, the diffraction picture will be smeared out, i.e., an effect similar to that corresponding to the Raman–Nath mode of interaction and diffraction (see Section 3.2.1).

The CLS effect is usually most pronounced for optical waves whose wavelength is roughly similar to the periods of the diffracting objects. The diffuse CLS observed here is different from the random diffraction grating effect (superposition of a large number of diffraction gratings with random amplitudes and phases) but, to some extent, is similar to the diffuse transmission of coherent light. Note that in mono-domain NLCs, the diffuse transmission due to anisotropic light scattering commonly does not follow the input light polarization and is polarization independent [60,61].

Further, a well-defined diffraction pattern as a sequence of light spots (peaks) horizontally localized (along direction *Y*) near the central peak can also be distinguished within the overall picture of the overlapping scattering/diffraction observed by the studied 5CB films. These features can be attributed to diffraction from a grating of a quasi-periodic structure characterized by multiple spatial frequencies. In this case, the most pronounced light peaks correspond to a higher periodicity for some spatial frequencies intrinsic to the diffractive structure. At a given wavelength, such a periodicity results in sharp diffraction peaks within the diffuse scattering field. Their localization and intensity depend on the configuration of the experiment. Similar light diffraction patterns in nematic liquid crystals with a positive dielectric anisotropy are well known [62].

**Figure 8:**
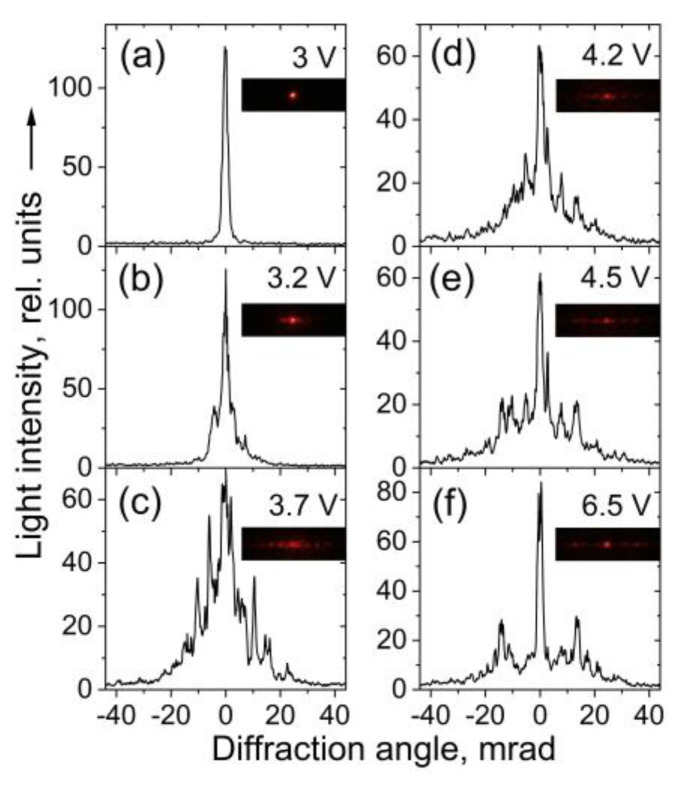
(**b**–**e**) Horizontal cross-sectional intensity profiles characterizing the CLS diffraction peaks (CLSDPs) observed by far-field imaging on a screen behind a planar cell with 25 μm-thick 5CB film upon DC electric field (*V_DC_* values indicated). The polarization of the incident He-Ne laser beam was parallel to the rubbing of the cell plates. The intensity profile (**a**) can be considered as corresponding to the non-scattered transmitted laser beam. The profile (**f**) is close to that of the pattern obtained by DC voltage-driven Fraunhofer diffraction from the 5CB film.

As found for the considered 5CB nematic films (Figure 8b–e) under the present experimental conditions, this effect (hereafter referred to as CLS diffraction peaks, CLSDPs) was most intense at *V_DC_* close to 3.7 V (Figure 8c), i.e., below the voltage *V_form_* = 4.2 necessary to form the wide LDs in the films (see Section 3.1.1). Also in this case, the spatial spread of CLSDPs reaches a maximum. Actually, Figure 8c presents a cross-sectional profile of a typical picture of CLS by the studied 5CB films, with the typical superposition of diffraction features as well as the transmitted central coherent beam.

When *V_DC_* ≥ *V_form_*, for example, *V_DC_* = 4.5 V (Figure 8e), the diffraction pattern transformation implies the formation of a real grating. With a further increase in *V_DC_*, diffraction patterns occur (Figure 8f) that are relevant to the diffraction effect corresponding to the morphology consisting of well-developed, wide-formed LDs discussed in Section 3.2.1, i.e., in this case, a director-modulation grating takes place, which results in clearly observed first-order (+1 and −1) diffraction intensity features (recall Figure 6b). The observed change in the diffraction pattern was consistent with the change in texture of the nematic 5CB film described in Section 3.1.

The field-induced CLSDPs, diffuse CLS, and DDS were present in both forward and backward directions, being considerably stronger in the forward direction (the same applies to the observed Fraunhofer diffraction pattern). Inspection of the spatially separated forward scattered/diffracted light forming these patterns shows that the optical noise signal due to scattering from the glass cell itself (as probed by an empty cell) does not contribute to their intensity. Also, any contribution from possible stray scattering from the optical elements of the experimental setup can be excluded, taking into account the geometry of the present experiment.

##### Fourier Analysis

**Figure 9:**
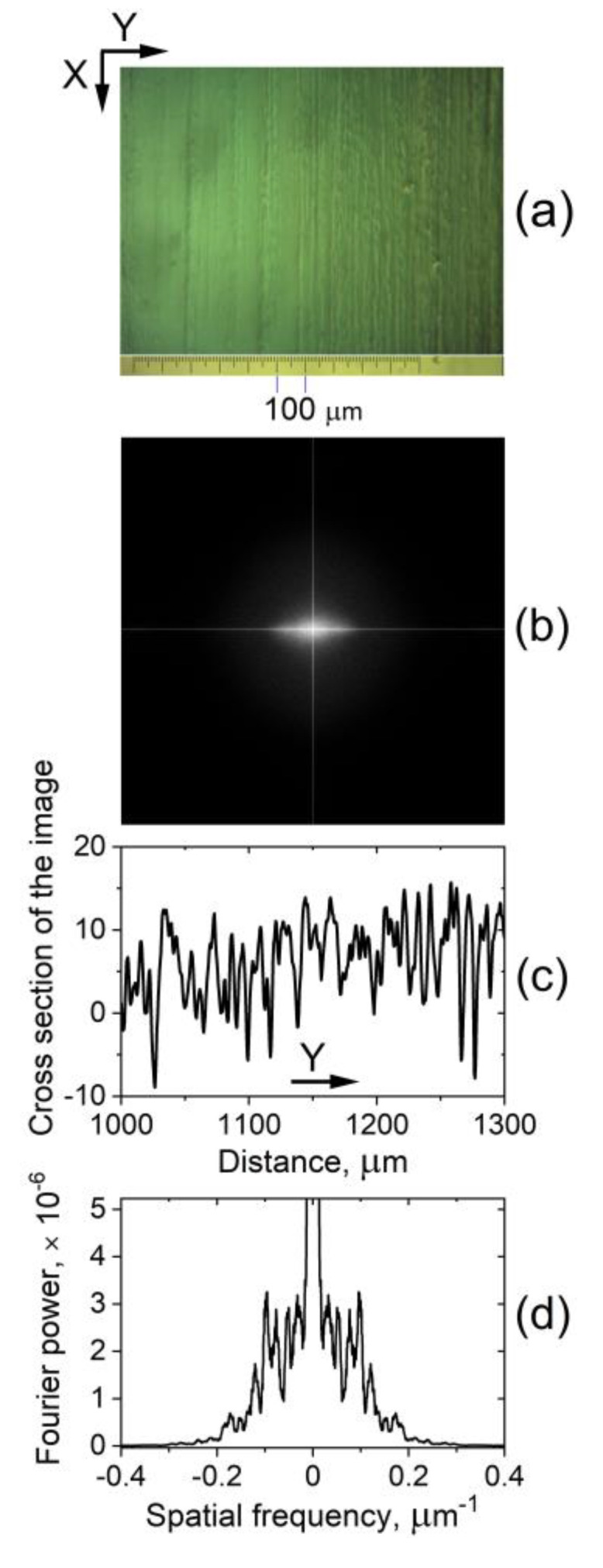
(**a**) Optical microscopy image of the texture formed in a 25 μm-thick planar-oriented 5CB nematic film at *V_DC_* = 3.7 V; (**b**) Fourier transform of image (**a**); Panel (**c**) represents an enlarged cross-sectional profile of texture image (**a**); (**d**) is Fourier transform of the whole cross-sectional profile of image (**a**).

It is worth noting that Fourier transforms of images of the fine parallel stripes in the studied planar-oriented 5CB nematic films under conditions when the wide LDs are not formed, i.e., at voltage *V_DC_* < *V_form_* applied to the cell (Figure 9a,b), resemble the observed CLS halo (Figure 7). On the other hand, the concentrated amplitudes around the central peak in the Fourier transform spectrum of a cross-sectional profile of such an image (Figure 9d) look like the intensity profile of CLSDP’s pattern. In fact, Fourier analysis allows a precise inspection of the texture change in the considered 5CB planar films upon low voltages *V_DC_* < *V_form_*. In contrast, the detailed monitoring of the field-modified narrow-striped texture (Section 3.1.2) with POM is difficult.

**Figure 10:**
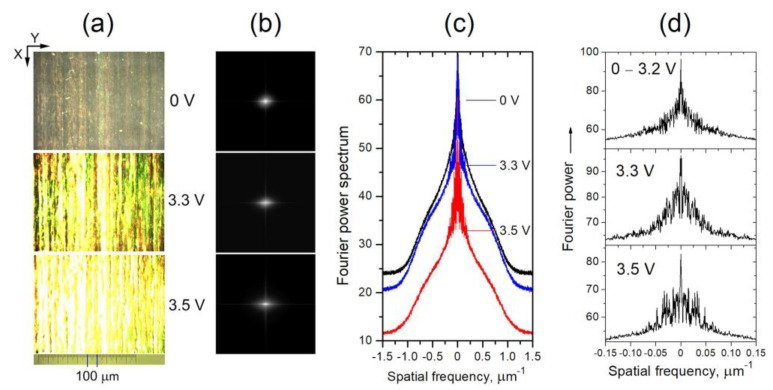
(**a**) Micrographs of the texture formed in a 25 μm-thick planar-oriented layer of nematic 5CB at two values of applied DC voltage. Slightly uncrossed polarizers (set along *X* and *Y*). The input light polarization was parallel to the initial alignment of the NLC (i.e., along *X*—the rubbing direction of the cell plates); (**b**) Fourier transforms of the images in (**a**); (**c**) Digitized images of Fourier transforms from (**b**); (**d**) Expanded view of (**c**) showing the central region of spatial frequencies.

As an example, Figure 10a shows micrographs of the examined nematic texture as viewed at two voltages below 3.7 V. Fourier transforms of the texture images (Figure 10b) reveal that in the voltage range from 0 to 3.2 V, there is indeed no change (the micrographs taken for the texture are identical). However, at *V_DC_* = 3.3 V a change in the texture is registered, as seen from the digitized images of Fourier transforms of the micrographs (Figure 10c,d). At higher voltages, e.g., *V_DC_* = 3.5 V, these profiles tend to match the spatial profiles of CLSDPs shown in Figure 8c. This suggests that the CLSDPs are generated by a narrow-striped microperiodic texture that acts as a grating, as described in Section Diffraction Pattern.

### 3.3. DC Voltage-Dependent Coherent Light Transmittance of 5CB Planar Films

The spatially periodic director-field modulation in nematic planar cells upon low-voltage static electric fields (discussed in Section 3.1), combined with the strong optical anisotropy that typically characterizes any NLC phase, causes the NLC layer to act as a diffraction grating (or quasi-diffraction grating) when illuminated with a monochromatic light beam (as presented in Section 3.2). Under appropriate conditions and certain circumstances, this effect can be used for EO control of the coherent light transmittance of the NLC films. 

#### 3.3.1. Central-Beam EO Behavior

**Figure 11:**
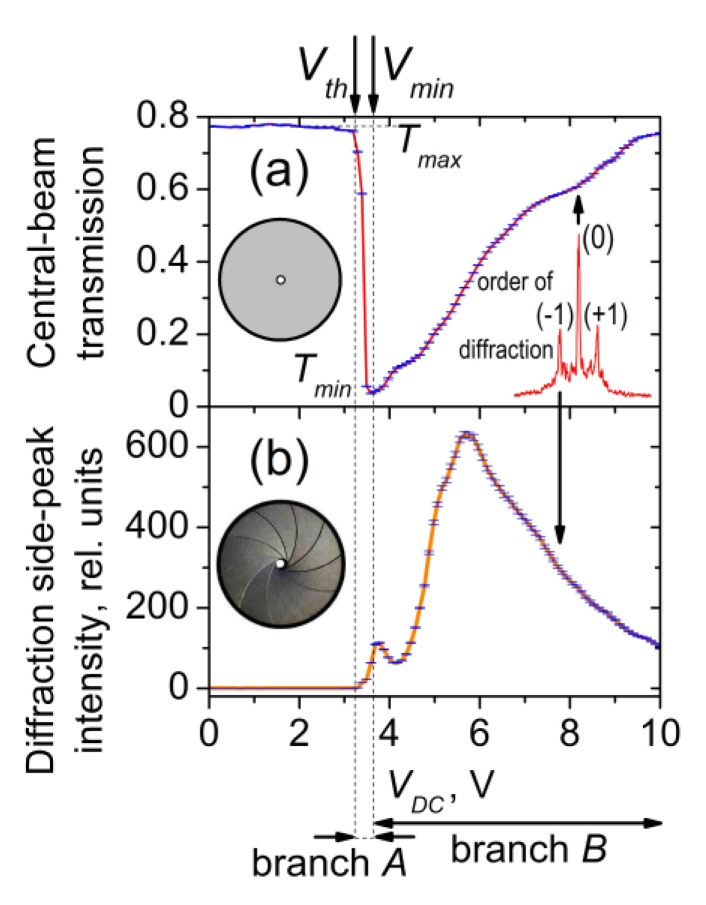
DC voltage-dependent intensity of (**a**) central part of the central beam, (**b**) side-diffraction peak of the diffracted-transmitted light, both measured behind a cell with 25 μm-thick planar 5CB film, in the far-field zone (85 cm distance from the cell to a photodetector with a pinhole). The inset in (**a**) illustrates a cross-sectional intensity profile of diffraction pattern, the same as the one shown in Figure 6b; the arrows indicate the measured light for the cases (**a**,**b**). The polarization of the incident He-Ne laser beam was parallel to the rubbing of the cell plates. The voltage values of the threshold (*V_th_*) and for a minimum transmittance (*V_min_*) are indicated on the top. At the bottom are marked the voltage ranges branch *A* and branch *B* of the *V_DC_*-dependent transmittance curve (**a**), noted in the text. The error bars (in blue) correspond to the standard deviation of the measured data (after 10 averaging).

Figure 11a presents the *V_DC_*-dependent intensity of the central radial part of the central beam transmitted through a planar 5CB cell. Such coherent light was separated by a pinhole in front of the measuring photodiode (recall Figure 1a). In this case, the separated coherent light was a superposition of coherent light transmission, zero-order Fraunhofer diffraction, and a small contribution of CLS. As seen in Figure 11a, at a well-defined voltage threshold *V_th_* (3.2 V), the gradual increase of *V_DC_* applied to planarly-aligned cells with 5CB results in a noticeable sharp decrease of the transmitted light intensity, from its maximum value (*T_max_*) to its minimum (*T_min_*). The latter was achieved at a *V_min_* = 3.6 V. A further gradual increase in *V_DC_* in the range of 3.7 V–10 V leads to a monotonical increase in the intensity of the central beam. For the two voltage ranges of the measured V-shaped transmittance curve, *V_DC_* from *V_th_* to *V_min_* and *V_DC_* > *V_min_* (hereafter referred to as “branch *A*” and “branch *B*”), respectively (Figure 11a), rather different EO behaviors take place. The reasons are also different.

Regarding the whole transmittance curve, the 5CB planar cells exhibited a fully reversible EO response under the conditions of the present experiment. When using the time-dividing scheme for measurements described in the Experimental Section, within the experimental uncertainty, the same V-shaped behavior was obtained by either increasing or decreasing *V_DC_*. Thus, the planarly aligned 5CB cells provide a low-voltage, hysteresis-free, controllable change in the transmitted coherent light that can be useful for practical applications. Notably, branch *A* is very suitable for efficient and low-voltage modulation of laser light.

It should be noted that the considered voltage-controlled coherent light transmittance curve depends on the polarization direction of the incoming laser beam as well as on (i) the texture illuminated; (ii) temperature; (iii) the beam incidence angle; and (iv) the wavelength of the incoming monochromatic light. This is reasonable since all these factors have an influence on the refractive index of the NLCs and their change with voltage applied to the nematic film. The EO behavior of the central-beam transmittance as it depends on (i)–(iv) is presented and discussed in the Supplementary Materials. If it is not specifically stated, a zero angle of incidence is implicit in this work. 

Besides the chemical structure of the NLC, the considered texture formations depend very strongly on the experimental conditions relevant to the LC cell (the NLC film thickness, the quality of the boundaries, their treatment, the alignment layers of the cell, the anchoring of the LC molecules at these layers, and other factors). Certainly, the polyimide alignment layers of the LC cells used here (as well as the way of rubbing and the rubbing’s geometry) have an important role in the formation of the DC-induced stripe domains as well as in the EO effect under study (a V-shaped curve). It should be taken into account that the polyimide material has a very high resistivity, much higher than that of the active NLC film. Hence, these layers consume some portion of the energy of the DC electric field applied to the LC cell to orient the 5CB molecules and modulate the NLC director. Such an effect of highly resistive orienting layers has been well established, e.g., reported in [63,64]. Due to such a low voltage drop (actually very low), one can expect that the V-shaped curve (and the corresponding *V_th_* and *V_min_* values) are slightly shifted toward the higher voltage values. Further, the contact resistance at the interface between polyimide-NLC may also have some impact on the EO effect considered here. However, investigations of the effect of the material of the alignment layers as well as the rubbing parameters of the LC cells are beyond the scope of the present work.

An important factor that may affect the registration of the EO response of the studied films is the detection geometry in the measurements. The curve shown in Figure 11a is relevant to the *V_DC_*-dependent central-beam transmittance of a planar 5CB cell when a small part of the transmitted light is selected for detection. This light comprises the central area of the central beam selected in our case through a pinhole with a small diameter (~1 mm). When a larger portion of the light around the center of the transmitted beam is registered, a weaker reduction of the film transmittance takes place. For instance, Figure S1b shows such *V_DC_*-dependent coherent light transmittance curves measured when the light is separated by the use of an iris diaphragm (centered around the *Z* axis, Figure 1a) whose opening size (circular aperture) is equal to the full diameter of the transmitted laser beam (at the location of the diaphragm). When one measures all the transmitted central-beam intensity, the value of *T_min_* is higher, i.e., the optical contrast ratio *T_max_*:*T_min_* decreases. Clearly, this is due to the contributions of the overlapping CLS processes (see Section 3.2.2) being partially included in the detection area. Being of importance for the application of the considered EO effect, light detection by measuring the beam center is most favorable. Also, the longer distance to the photodetector is profitable.

#### 3.3.2. EO Behavior of Coherently Diffracted and Scattered Light

##### Voltage-Dependent Fraunhofer Diffraction

The intensity of the observed diffraction side-peaks of the triplet split of coherent light resulting from Fraunhofer diffraction by the wide-formed LDs (Section 3.2.1) also depends on *V_DC_* (Figure 11b). Reasonably, this is due to the change in optical anisotropy modulation caused by the applied electric field. Since the present case is not purely diffractive, the amplitude transmittance function of the diffractive medium and its variation with *V_DC_* cannot simply be deduced from the measured spatial distribution of the diffracted light intensity. This is an inverse problem that would otherwise have to be solved routinely using theory and indirect methods. Generally, the cross-sectional profile of a texture image (a micrograph taken by transmission, such as the one shown in Figure 2) represents the modulus of the complex amplitude transmittance function, which is a spatially averaging product of the transmittance coefficient and its complex conjugate.

Like the *V_DC_*-dependent central-beam transmission (Section 3.3.1), the DC voltage-controlled diffracted light through the electrically-induced LDs in the considered planar nematic films was fully reversible at ascending or descending *V_DC_*. It should be mentioned that the decrease in light intensity in the first diffraction orders is related to energy conversion and redistribution to the zero-th order [42]. 

##### Voltage-Dependent CLS

Regarding the *V_DC_*-dependent spatial and light intensity changes of field-induced diffuse CLS, by increasing *V_DC_* in the voltage range from *V_th_* to *V_form_*, the size of the CLS halo was gradually increased (see the photo series Pics1 in the Supplementary Materials). The same applies to the DDS stripe, but in the voltage range from *V_th_* to *V_min_*. The asymmetry of this pattern of CLS is like that for light scattering from NLC director anisotropy fluctuations, which follows a simple rule: the scattered intensity is highest in the direction orthogonal to the polarization direction of the incident light. This has been well established for NLCs and, more specifically, for 5CB [60]. Reasonably, the horizontal-to-vertical asymmetry of the spatially localized CLS pattern behind the examined planar cells with 5CB (CLSDPs and DDS, see Section Diffraction Pattern) may be considerable.

At the value *V_form_* (4.2 V), the transversal light distribution of diffuse CLS (both circular-chaped halo and DDS stripe) reaches maximum intensity (can be seen in the photo series Pics1 in the Supplementary Materials), and the halo is transmuted into a circular ring from a diffracted light cone around the central beam of transmitted light (recall Figure 6a). When *V_DC_* is above *V_form_* and gradually increases, the CLS is weakened, and the intensity of the arising side-peaks of the triplet Fraunhofer diffraction pattern is enhanced (photo series Pics1 and Pics2 in the Supplementary Materials). By further increasing *V_DC_*, the intensity of the CLS patterns is gradually decreasing simultaneously with the diminishing of the first-order diffracted laser beam intensity. At higher *V_DC_* (*V_DC_* > 7 V), when the electrically-induced hydrodynamics in the 5CB cells are enhanced, both Fraunhofer diffraction and CLS are greatly reduced. Still, some of the light behind the cells is diffracted and scattered by fragmented LDs (which is a superposition of field-induced periodic and aperiodic modulation of optical anisotropy).

#### 3.3.3. Polarization Dependence 

**Figure 12:**
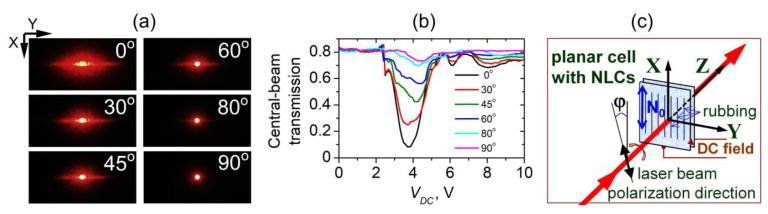
Changes in the diffraction pattern (pictures taken on a transversal screen) behind a 25 μm-thick planar cell with 5CB at *V*_DC_ = *V_min_* = 3.7 V (**a**) and *V_DC_*-dependent central-beam transmittance of the same film (**b**) for various angles φ of the He-Ne laser beam polarization toward the rubbing direction of the cell: 0°; 30°; 45°; 60°; 80° and 90°, under the same other experimental conditions. Normal incidence of the laser beam (**c**).

The sharp reduction of the intensity of the laser beam transmitted through 5CB planar films presented in Section 3.3.1 is polarization sensitive. Figure 12a illustrates the change in diffraction pattern behind the 5CB cell depending on the direction of polarization of the incoming laser light at a fixed value of *V_DC_*. Figure 12b shows the corresponding change in the *V_DC_*-dependent central-beam transmittance of the films measured under identical experimental conditions.

As seen from Figure 12b, the reduction effect is strongest when the polarization of the incident laser beam is parallel to the rubbing direction of the examined cells (direction *X*, see Figure 12c), i.e., to obtain maximum effect, the electric field of the incident plane-polarized light wave must be parallel to the initial (zero-field) director orientation (**N_0_**) and the field-induced LDs. This is consistent with the geometry of the spatially periodic orientation pattern *V*_DC_-induced in the studied 5CB planar films. In the case of parallel **N_0_** and laser beam polarization, the laser beam propagating through the films most effectively experiences static nematic director deformations. In turn, the diffraction grating induced in the films by the applied DC electric field does not respond to light polarized orthogonally to the direction **N_0_**. 

**Figure 13:**
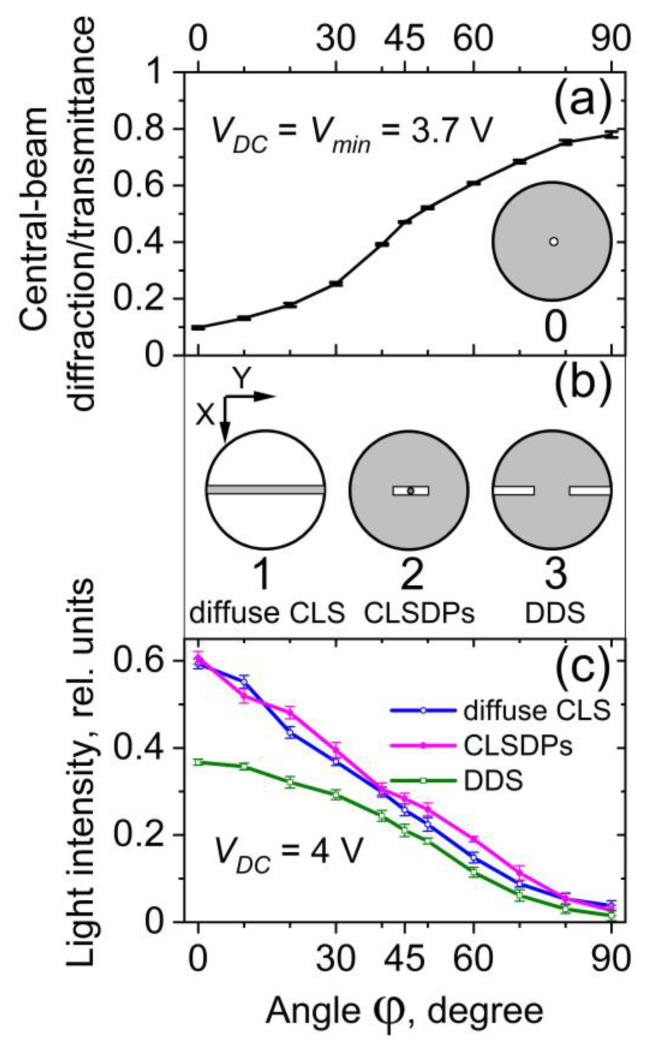
The intensity of central-beam diffraction/transmission (**a**) and CLS (**c**) measured for a 5CB planar cell as a function of the angle φ between the He-Ne laser beam polarization direction and the rubbing of the cell, under the same other experimental conditions. Error bars correspond to the standard deviation of the data obtained after 10^4^ averaging (done during 30 s). (**b**) Illustrations of the spatial filtering performed—the blocked area on the collecting spherical lens is colored in gray; the distance cell-to-lens was 25 cm.

Figure 13 compares the dependences of both central-beam coherent light transmission and of CLS against the angle φ between the direction of the polarization of the incident laser beam and the rubbing direction of the 5CB cell (the initial orientation of the nematic director, **N_0_**). The polarization-dependent central-beam transmission was measured at *V_DC_* = *V_min_*, according to the scheme shown in Figure 1a, using a pinhole as a spatial filter (Figure 13a, filter 0). It should be remembered that for the 5CB films understudy, a small portion of CLS always accompanies the central light beam and enters its measured radial zone.

The polarization-dependent intensity changes of CLS patterns behind a 5CB planar cell at a fixed voltage (*V_DC_* = 4 V) are given in Figure 13c. In these measurements, the different CLS patterns were separated by spatial filtering of light on a collecting lens, as shown in Figure 1b. In each of these cases, the *V_DC_*-dependent intensity of the forward scattered/diffracted light was recorded while blocking the central beam of transmitted light. The diffuse CLS (the halo and its transformation into a circular diffraction ring at higher *V_DC_*) was measured by using a narrow (3 mm) horizontal blocking stripe (Figure 13b, filter 1). In this way, the horizontally located diffraction/CLS patterns were rejected, i.e., CLSDPs and DDS. The accurate and complete separation of CLSDPs and DDS is a difficult task, but the applied spatial filters are acceptable solutions for their individual measurements (Figure 13b, filters 2 and 3, respectively). 

It is seen from Figure 13 that, in contrast to the DC electric field-controlled central-beam transmittance of the 5CB planar cell, the light intensity of the components of field-induced CLS from the cell (diffuse CLS, CLSDPs, and DDS) was maximum when the polarization of the incident coherent plane optical wave was along the rubbing of the cell plates and was reduced when the input polarization was rotated towards the orthogonal direction.

### 3.4. EO Control of Coherent Light Transmission through LDs in Nematic Films—Physical Mechanism

The shift between the minimum of the light transmittance curve shown in Figure 11a and the maximum of the curve in Figure 11b implies that the sharp decrease in the intensity of the central beam of coherent light transmitted through the studied 5CB planar films has to be related to a physical process other than the diffraction splitting of the laser beam due to Fraunhofer diffraction (discussed in Section 3.2.1). A detailed inspection indicated that the observed strong reduction effect at *V_th_* < *V_DC_* < *V_min_* (i.e., branch *A* in the central-beam transmittance curve, Figure 11a) can be connected with the DC electric field-induced CLS from grating-scattering *V_DC_*-induced microperiodic narrow-striped texture in these nematic films (Section 3.1.2). As mentioned in Section 3.1.1 and Section 3.2.1, the wide-formed LDs in the 5CB films under study occur at a DC voltage *V_form_* = 4.2 V, i.e., higher than *V_min_*. Accordingly, in the voltage range *V_th_* < *V_DC_* < *V_min_* the CLS should not compete with the *V_DC_*-induced Fraunhofer diffraction from the films.

**Figure 14:**
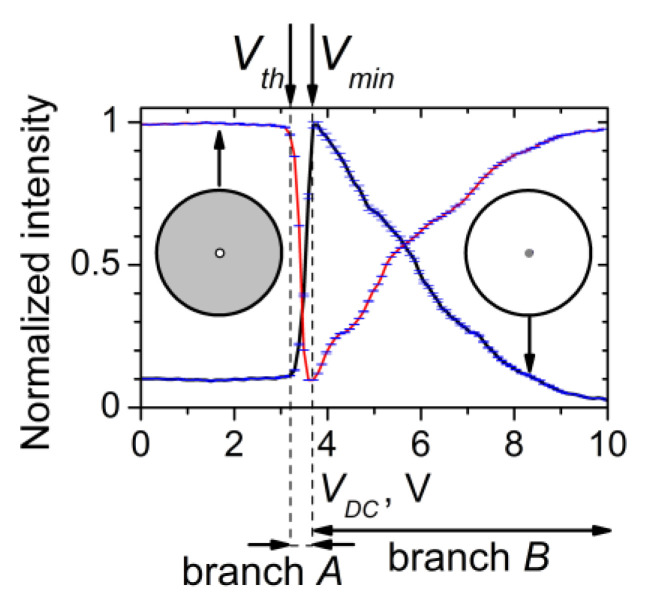
*V_DC_*-dependent intensity of central-beam transmitted light (shown with circles) and scattered/diffracted coherent light (line), measured separately in the near-field zone at a distance of 15 cm behind a planar cell (thickness 25 μm) with 5CB. The polarization of the incident He-Ne laser beam was parallel to the rubbing of the cell plates. Both curves were obtained under identical experimental conditions, except for the spatial filtering of the light. The spatial filters employed are illustrated by inset sketches of the blocked area (colored in gray) on the collecting spherical lens. The central-beam transmitted light was measured using an iris diaphragm with an aperture diameter equal to the diameter of the input laser beam and centered on that beam. The scattered/diffracted coherent light was registered by blocking the central beam using a circular aperture with the same diameter as that beam. The error bars (in blue) correspond to the standard deviation of the measured data (after 10 averaging).

When only the central beam was blocked and the total intensity of forward diffracted and scattered light from the 5CB film was measured, the recorded curve was the reciprocal of that measured for the central beam intensity (Figure 14). These opposite EO behaviors indicate the close correlation between the CLS/diffraction and the central-beam diffraction/transmission (the latter being still slightly influenced by CLS/diffraction). These optical processes are a -counter-pair. They are coupled and controlled by the electrically-driven reorientation of the 5CB molecules towards the direction of the DC electric field (which is also the direction of the incident laser beam, Z, Figure 1b) and orthogonal to the initial orientation of the director along the rubbing of the cell plates (the direction *X*). 

Being influenced by CLS and diffraction, the coherent light transmission in our case cannot simply be modeled as the transmittance of the nematics, considered only as birefringent media [65,66]. The propagation of an optical beam and its diffraction by a grating formed in such media due to the periodic inhomogeneity of their optical refractive index need more complex analyses and sophisticated interpretation [67]. As with other bulk NLCs, light scattering from 5CB nematic films is generally due to the anisotropy of the index of optical refraction (birefringence, Δ*n*) of the NLC material. With a plane monochromatic optical wave of wavelength λ passed through a nematic film of thickness *d*, an optical phase difference (shift) is induced that, at normal incidence of the wave, is expressed as δ = 2π*d*Δ*n*/λ. The optically-induced δ can modulate the optical wave interacting with the nematic film. When an electric field is applied to the nematic film, a field-induced Δ*n* occurs, expressed as Δ*n = n*_e_(*E,ψ*) − *n*_o_, where *n*_e_ and *n*_o_ are the extraordinary and ordinary index of refraction, respectively, *E* is the electric field intensity, and *ψ* is the spatial angle between the nematic director and the direction of the incident optical beam. Thus, the corresponding field-induced δ depends on the orientation of the NLC molecules by the applied electric field.

In our case, the optical anisotropy Δ*n* induced in NLC planar films by DC voltage *V_DC_* is spatially modulated and expressed as periodic LDs—an electrically formed microperiodic array. The spatially modulated anisotropy and the *V_DC_*-induced change in orientation of the nematic director result in microperiodic spatial patterns of phase shift—an electrically-induced optical phase grating whose orientation is electrically controlled. Due to the positive dielectric anisotropy of the 5CB molecules, they are forced by the applied electric field to orient themselves relative to the field direction (in our case, along the Z axis, Figure 1). Thus, the CLS and diffraction from the studied nematic films are electrically induced (via the electrically induced LDs texture in them) and electrically driven by the electrically driven reorientation of the nematic director (the local optical axis).

**Figure 15:**
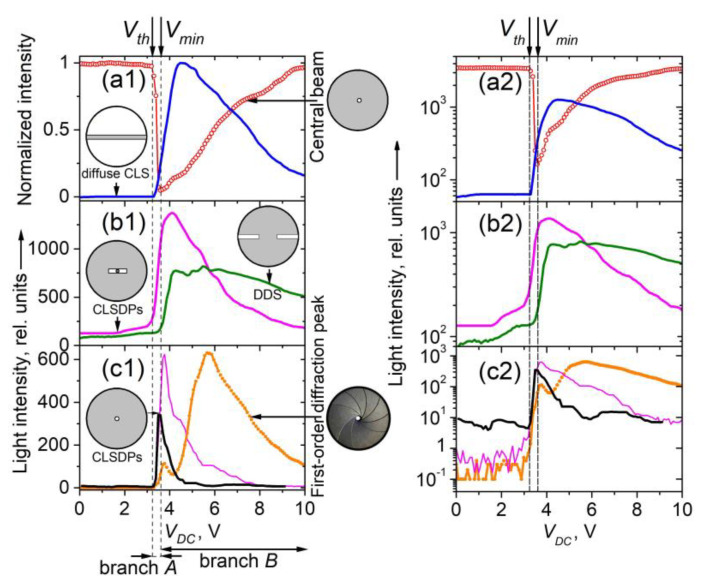
*V_DC_*-dependent intensity of light behind a planar cell with 5CB film (thickness 25 μm): (**a**) central beam of diffraction-transmission and diffuse CLS; (**b**) CLSDPs and DDS; (**c**) the first-order diffracted light and diffracted light measured at two intermediate locations between the central peak and the first diffraction order (drawn with a thin and bold line). They, as well as the central beam of diffraction-transmission of coherent light, were measured in the far-field zone (85 cm distance to the cell, photodetector with a pinhole). In the measurement of diffuse CLS (**a**), CLSDPs and DDS (**b**), the distance cell-to-lens was 25 cm. In all cases, the polarization of the incident He-Ne laser beam was parallel to the rubbing of the cell plates. The performed spatial filtering is illustrated by inset sketches of the blocked area (shaded in gray) on the collecting spherical lens (see also Figure 1b). In the right—the same as in the left, but in logarithmic scale for the light intensity. At the bottom are marked the voltage ranges branch *A* and branch *B* of the *V_DC_*-dependent transmittance curve for the central beam. The measurement error was 1.5–2.5%.

By measuring the integral intensity of CLS/diffraction, one cannot specify what is the origin of the sharp decrease in the intensity of the central beam, i.e., branch *A* of its *V_DC_*-dependent transmittance curve. In order to be compared, Figure 15 presents the *V_DC_*-dependent intensity curves for the transmitted light corresponding to the coherent optical processes considered above (Fraunhofer diffraction and diffuse CLS), separately measured after spatial filtering of the light behind the 5CB planar cell. Figure 15a shows the intensity of the measured light due to forward diffuse CLS as compared with the intensity of the central beam of diffraction/transmission. On the other hand, Figure 15b reports the intensity of the selected forward horizontal diffraction/CLS pattern—CLS diffraction peaks (CLSDPs) and the diffuse-diffraction spectrum (DDS) (see Section 3.2.2). Figure 15c presents the *V_DC_*-dependent intensity measured for selected forward first-order diffracted light due to Fraunhofer diffraction. In this measurement in the far-wave field, the coherent light was separated by an iris diaphragm, whose circular aperture was equal to the size of the first-order diffraction pattern (peak). It should be noted that in this case, the unavoidable contribution of CLS/diffraction results in a complex curve (Figure 15c). For completeness, in Figure 15c, the *V_DC_*-dependent intensity of two other bright peak-like patterns is also given, each located exactly between the central peak and the +1 or −1 order of Fraunhofer diffraction from the 5CB film. These *V_DC_*-induced light peaks were measured separately in the same way in the far-field zone. Most probably, they are diffraction peaks from the CLSDPs sequence. 

Comparing the data in Figure 15 for the distinguished coherent optical processes electrically driven through the *V_DC_*-induced LDs in the measured 5CB planar cells, as well as the dynamic ranges of the intensity changes of their EO behaviors, one can conclude that the most active optical process that competes with the coherent transmission of the central beam in the considered small voltage range *V_th_* < *V_DC_* < *V_min_* is the CLS expressed as CLSDPs. More strictly, both CLSDPs and diffuse CLS from the narrow parallel stripes (as a fine microperiodic grating of textural LDs) are related to the sharp decrease (branch *A*) of the intensity of the laser beam passed through the planar 5CB films examined here.

As for the gradual decrease in CLS intensity at voltages above 4 V (Figure 14 and Figure 15a,b), this is related to the formation of wide LDs in the studied 5CB films and the *V_DC_*-induced Fraunhofer diffraction from them, respectively. Because of the positive dielectric anisotropy of the 5CB molecules, their electrically driven orientation tends to a homeotropic alignment of the initially planar 5CB nematic films. At increasing *V_DC_*, this process, followed by quenching of LDs associated with a transition to a state of electrohydrodynamic instability and random motion inside the nematic films, leads to a diminishing of modulated anisotropy and the efficiency of the formed optical phase grating experienced by the incoming linearly polarized laser beam, and hence to reduced laser scattering and diffraction. This results, namely, in the decreasing wings of CLS-related curves, seen in Figure 14 and Figure 15. At *V_DC_* > 9 V, the beam intensity spatial profile tends to its initial shape, i.e., the scattering and diffraction disappear. Thus, the field-induced quenching of the microperiodic director-modulation textures can adequately explain branch *B* of the EO characteristic curve of the central beam of diffraction/transmission.

### 3.5. Enhancement of the Optical Contrast Ratio of Coherent Light Transmittance of Nematic Films, Electrically-Controlled by Microperiodic Director Modulation

Most likely, other NLCs can also exhibit DC low-voltage scatter spatially periodic director modulation patterns and hence suitable coherent scattering and diffraction by achieving a complex balance of various system parameters that control the formation of LDs (the periodic director modulation) and thereby controlling the complex transmittance function that determines the EO response of NLC planar films. An effective way to improve the optical contrast ratio in this case can be doping methods, for example, by including suitable nanoparticles (NPs). As practice shows, the doping of NLCs with even a small amount of NPs affects almost all important properties of the nematic materials (for example, [68,69]). In particular, metal NPs can considerably modify the texture, optical, and EO properties of such NLC-based nanocomposites compared to the host NLC material, e.g., 5CB and other cyanobiphenyls [69,70,71,72]. Moreover, there are reports showing the formation of periodic structures and stripe patterns in 5CB nematic doped with gold nanoparticles (AuNPs), which are of special interest for metamaterials and the fabrication of tunable photonic and communication devices [73,74]. Also, such nanocomposite materials can exhibit novel EO effects. The search for enhancement of the optical contrast ratio of the electro-optically controlled light transmission in nematics and related composites is a challenge.

**Figure 16:**
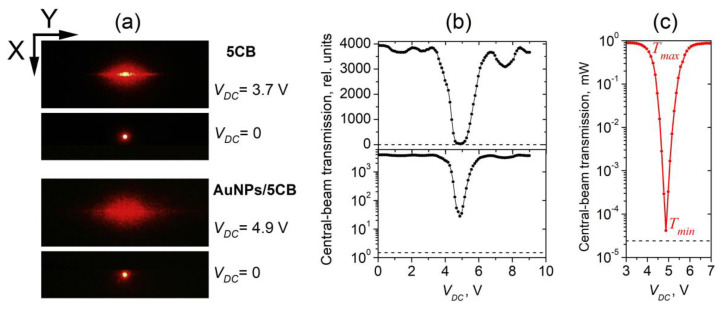
(**a**) Representative examples of maximum diffractive/CLS spread and laser beam intensity reduction effects resulting from He-Ne laser beam propagating through identical cells with 25 μm-thick planar nematic films of 5CB and AuNPs/5CB upon DC electric field. The pictures were taken for far-field light intensity patterns displayed on a transversal screen behind the cells. The laser beam polarization direction was parallel to the rubbing of the cell plates; the other experimental conditions were also the same. The circular beam shapes corresponding to the zero-field transmission are given for comparison; (**b**) DC voltage-dependent intensity of He-Ne laser beam passed through a 25 μm thick planar cell with a composite of 0.5 wt.% gold nanospheres in 5CB nematic. The transmitted laser beam is measured in the far-field zone (85 cm distance to the cell, photodetector with a pinhole). The curve is given in both linear and logarithmic scales. The detection limit (the dark-current signal) of the apparatus is shown with a dashed line; (**c**) As in (**b**), but measured with a higher dynamic range of photodetection.

Some efforts in this regard are focused on investigating metal NPs and hybrid metal-polymer nanostructures that can be quite effective as additives to nematics. For instance, a large reduction (~20 dB) in a short voltage range ~1.5 V) of coherent light transmission of a He-Ne laser beam has been obtained by mixtures of 5CB and 12 nm-diameter gold nanospheres at a relatively low concentration of 0.5 wt.% [41]. These AuNPs were capped with a ca. 10 nm-thick polymer layer [41]. Modifying the NLCs 5CB by adding such NPs, the nematic state holds, and the dynamic range (optical contrast ratio) of the coherent light intensity change can be markedly enhanced. Compared to the reduction effect found with identical planar cells with 5CB nematic discussed in Section 3.4, the improvement was at least one order of magnitude (Figure 16b). Such an electrically activated effect of AuNPs/5CB composite films upon low-voltage static electric field results from the larger spatial spread of light in a direction orthogonal to the polarization direction of the incident laser beam (in the present case—horizontal spread, along the *Y*-axis, Figure 16a).

As in the 5CB films, the complex interplay between the electrically induced light scattering and diffraction is determined by the texture that is dominant at the corresponding value of the voltage *V_DC_* applied to the AuNPs/5CB film. Again the field-induced narrow-striped LDs fine-stripe microperiodic texture and the diffusive-diffractive CLS strongly decrease the intensity of the laser beam transmitted through AuNPs/5CB films, as in branch *A* of the V-shaped *V_DC_*-dependent coherent light transmittance curve for 5CB films. There is, however, an important difference. For 5CB films, the increasing coherent light transmission at *V_DC_* > *V_min_* is mostly due to Fraunhofer diffraction from the wide-formed LDs (Section 3.2.1). At such voltage values, these LDs become more pronounced (Section 3.1.1). They are developed near the cell plates and over the rubbing-induced narrow-striped LDs [1,51,53]. By further increasing *V_DC_*, flexo-dielectric walls in 5CB films replace the rubbing-induced surface texture. Accordingly, Fraunhofer diffraction replaces the scattering. In contrast, the EO effect observed with AuNPs/5CB planar nematic films is solely due to rubbing-induced surface texture. In this case, the strong minimization of coherent light transmission at *V_min_* results from the specific angular spread of scattered-diffracted light on both sides outside the central beam at a certain *V_DC_* value [41].

The contrast ratio *T_max_*:*T_min_* at *V_DC_* = *V_min_* is limited due to the same CLS. The light due to CLS appears as optical noise (coherent background) for the central beam signal, although the divergence of CLS is much larger than that of the central beam of transmission-diffraction. Due to this limitation, the contrast ratio depends on the measurement/detection scheme. Thus, the V-shaped curve for the central-beam transmission is closely related to the detection geometry and both the sensitivity and dynamic range of the photodetector. To achieve a maximum effect, the iris diaphragm in front of the photodetector should be correctly adjusted (centered around the direction of the output laser beam) and opened in the proper way in order to register only the central part of this beam. Furthermore, the polarization vector of the laser beam has to be exactly parallel to the rubbing of the cell (see Section 3.3.3), and the laser beam must be incident normally to the cell with the nematic film (Section S1.3 in the Supplementary Materials).

The dominant longitudinal texture patterns in the AuNPs/5CB planar cells viewed by POM (including observations at DC voltage in the range from 4 V to 7 V) were composed of densely spaced regular narrow (micrometer-wide) parallel stripes, oriented along the rubbing direction of the cells [41]. The dispersed AuNPs, even at the relatively low concentration of 0.5 wt.%, prevent the formation of voltage-induced wide LDs (flexo-dielectric walls) observed in the planar cells with pure 5CB. This is due to the charge-trapping effect of the polymer-capped Au metal nanospheres, which leads to ion depletion in the bulk of the 5CB nematic films [41,71] (for a thorough conceptual review of ion-trapping effects from nano-objects in LCs and related phenomena, one can refer to Garbovskiy and Glushchenko [75]). 

In contrast to the 5CB films, the planar AuNPs/5CB composite films allow a full deflection of the light energy outside the beam center and, thereby, a complete EO minimization of the transmitted laser beam intensity in the far-field zone (see videos 1, 2, and 3 in the Supplementary Materials). It should be noted that the effect due to CLS/diffraction owing to the included AuNPs is much stronger than the decrease in the NLCs transmittance due to oscillations by the ordinary voltage-modulated birefringence (e.g., [66,76]). Hence, such nanocomposite nematic films are of great interest for EO applications, as discussed in the next section.

### 3.6. Applicability of Coherent Light Transmission Electrically Controlled by Spatially-Periodic Director Modulation in Nematic Films under Low-Voltage Static Electric Field

V-shaped voltage-dependent optical transmission (also termed “transmissive U-shaped EO switching”) is well known for LC structures (smectic and ferroelectric) [77,78,79,80,81,82,83]. The specific EO control of coherent light transmittance of nematic films by scatter spatially periodic director modulation in them can be used for new modes of scatter-based EO applications exploiting the spatial patterns of optical phase shift induced in NLC films upon low-voltage DC electric fields. As presented in Section 3.5, AuNPs/5CB composites in planar-orienting cells under low-voltage static electric fields exhibit a large and sharp reduction of transmitted laser beam intensity. The registered dynamic range of this EO effect depends on the sensitivity limit and dynamic range of the photodetector and measurement unit. Practically, in this way, one can achieve an extremely high optical contract ratio *T_max_*:*T_min_*–higher than 10^4^ (Figure 16c)—if a photodetector with such a large dynamic range is used. Such 100% efficiency can only be compared to that provided by the light deflection effects used by LC beam steering devices (for a thorough review, one can refer to, e.g., He et al. [84]). However, the LC deflectors typically require relatively high operating voltages at comparable thicknesses (25 μm) of the LC films, as well as the application of an AC electric field [84].

As noted in Section 3.5, AuNPs/5CB planar films can allow a full scattering of the light energy out of the beam center and thereby complete EO minimization of the transmitted laser beam intensity. Basically, the EO result of their operation is like that of the NLC-based polymer-dispersed liquid crystal (PDLC) devices [85,86,87] although the operation mechanism is different. The PDLCs operate through an electrically controllable dielectric reorientation of the LC molecules in micro- or nano-sized droplets. By applying an AC electric field of appropriate magnitude, the PDLCs are switched from the OFF-state (translucent state) to the ON-state (transparent state). The problem with PDLC devices is their relatively low optical contrast ratio, since it is practically impossible to eliminate optical scattering in the direction of the laser beam passing through the PDLC film. By using high-contrast PDLCs for high-performance EO shutters/modulators, this ratio can be as high as 30–50 [85,86,87], but values of 200–300 have also been reported for PDLCs with a special composite design [88,89,90]. Various approaches used to solve the problem of the low contrast of PDLC films require the application of a high control voltage. Alternatively, low-voltage interference effects in microscale single-layer PDLCs with large-sized NLC droplets have been proposed to increase the optical contrast ratio of coherent light transmission, making them suitable for tunable modulators of laser light [91]. 

However, by the reduction effect for electro-optically controlled coherent light transmission through LDs in NLC films considered in the present work, can achieve a much higher optical contrast ratio *T_max_*:*T_min_*. As with PDLCs, in this case, the laser light modulation is also based on electrically controlled light scattering and also depends on the optical properties of the NLC as a function of temperature and light wavelength (e.g., [43,92] for 5CB). The advantage of the EO effect of DC field-induced scatter/diffractive microperiodic textures of phase-shift regular spatial patterns in NLC films studied here is the strong reduction of light scattering in the optical path of the transmitted laser beam. Moreover, due to a splitting of the transmitted laser beam laterally in the direction perpendicular to the rubbing of the cell plates as well as due to the spatial shift of the scattered light to the periphery in the same direction (*Y*), the light can even be self-removed from the optical path of the transmitted laser beam, as reported for AuNPs/5CB planar nematic films under a static electric field [41].

Having a very sharp dip (fwhm less than 1.5 V), the specific low-voltage V-shaped curve of DC voltage-dependent coherent light transmittance of planar nematic films (Figure 16b,c) is certainly of interest to practice. For example, these films can be applied in the field of process control to stabilize various processes through electric feedback, which is a common application of V-shaped electrical characteristics. The state of the minimum laser beam transmission maintained by *V_DC_* = *V_min_* applied to the cell is very sensitive to any change in this voltage level. Accordingly, the nematic film can respond to very small voltage changes, e.g., by ±0.01 V. 

Despite the complexity of the EO response of NLC media to a static electric field, a periodic switching regime based on the EO effect described here has the potential to be implemented for control functions. The effect is easily usable, but the EO switching by the studied 5CB planar films needs a time of 1 s in the configuration for measurements in the far-field zone (Figure S5a) or 20 s in the near-field zone (Figure S5f) to achieve a stable recovery of the stationary state. Hence, the maximum EO modulation frequency (repetition rate in the pulsed regime) is limited and should be less than 1 Hz or 0.05 Hz, respectively. Thus, the considered low-voltage DC electric field-driven modulation via spatially periodic director-field modulation in nematic planar cells can be used for EO control of relatively slow processes. Also, the instability and long-time dielectric relaxations inherent in nematic films, especially at DC voltages in the range of branch *A* of the V-shaped coherent light transmission curve (see Figure 11), may limit the applicability of this EO effect for laser intensity modulation by DC repetition pulses. Notice that this applies to the time response of planar nematic films of both 5CB and AuNPs/5CB composites [41].

The applicability of the specific EO response of planar nematic films, e.g., 5CB and AuNPs/5CB considered here, can be extended owing to the possibility that the scattering/diffractive director-field modulation pattern induced in them with DC voltage can be rapidly erased by joint AC voltage [41]. The value of the latter is also low, comparable to the DC voltage driving the scattering/diffraction effect through modulated anisotropy [41]. The erasure of the *V_DC_*-induced optical phase grating (and thus the V-shaped transmittance of planar nematic films) can be relatively fast, e.g., within 0.1 s or less, depending on the strength of the externally applied AC electric field ([41]). As compared to 5CB films, AuNPs/5CB nematic films can offer faster and more stable EO modulation.

Finally, the V-shaped dependence of laser light transmittance determined by spatially periodic director modulation in NLCs can be used in low-voltage sensors and various optoelectric techniques for sensitive detection of weak dynamic electric fields. This option is useful for the detection/monitoring of events relevant to military, geo-acoustic, and biomedical applications.

## 4. Conclusions

Stationary longitudinal domains (LDs) formed in planar-oriented nematic films under a low-voltage DC electric field lead to coherent light scattering and diffraction. In this way, such films can enable efficient control of the transmission of coherent light through them. The effect is maximal when the polarization of the incident light wave is along the orientation direction of the field-induced LDs, i.e., parallel to the initial (zero-field) orientation of the nematic director.

At a DC voltage from zero to 10 V, two sets of electric field-induced regular LDs are observed in 25 µm-thick nematic 5CB films in planar cells: small-period (less than 10 µm) and large-period (~60 µm) LDs, both of which are of flexoelectric origin. The first type of LDs are induced by the orientation rubbing of the alignment layers of the cell; the second type of LDs are flexo-dielectric walls and take place at a voltage higher than a well-defined threshold value. As a result of each of these two kinds of field-induced periodic modulation of the nematic director, a field-induced spatial modulation of the optical phase in the plane of the nematic films arises due to the optical anisotropy modulation.

It is proven here that the microperiodic narrow-formed LDs induced by a low-voltage (~4 V) DC field can produce a sharp and large reduction of the intensity of a laser beam transmitted through the studied nematic films. By incorporating specific additives, e.g., NPs, the optical contrast ratio achievable with this EO effect can reach 10^4^, which is unattainable with conventional NLC-based devices for active light control. In this way, one can switch or greatly modulate the intensity of a laser beam propagating through planar nematic films if the beam polarization is parallel to the initial orientation of the nematic director.

Furthermore, the field-induced regular wide director-modulation spatial patterns of optical phase shift in planar nematic films can also be used for DC voltage-controllable amplitude modulation of coherent light. In planar 5CB films at DC voltages from 4 V to 10 V, the intensity of the diffraction splitting of the transmitted laser beam can be almost linearly commanded by the applied DC field, and such EO behavior is also of practical significance.

The nature of the observed EO effects is elucidated. The distinct voltage regions corresponding to the involved coherent optical processes (coherent light scattering and diffraction from DC voltage-induced gratings of optical anisotropy modulation) are exactly specified. The relationship of these processes to the DC voltage-dependent coherent light transmittance of planar nematic films is defined.

Of relevance to EO applications, advanced high-performance nematic materials (e.g., NLC-based nanocomposites or hybrid materials) can be designed that have reversible low-voltage EO scattering/diffraction responses through field-induced microperiodic director modulation and field-controlled director orientation. Such electrically controlled optical phase gratings and non-absorbing light diffusers can be adopted for various micro-optic and photonic applications.

## Data Availability

The data presented in this study are available on request from the corresponding author.

## Supplementary Materials

The following supporting information can be downloaded at: https://www.mdpi.com/article/10.3390/ma16176014/s1, Figure S1: DC voltage-dependent laser beam transmission through 5CB film; Figure S2: DC voltage-dependent laser beam transmission through 5CB film at various temperatures; Figure S3: DC voltage-dependent laser beam transmission through 5CB film at various angles of incidence, Figure S4: DC voltage-dependent laser beam transmission through 5CB film at various wavelengths, Figure S5: Temporal behavior of DC voltage-dependent laser beam transmission through 5CB film, Figure S6: Optical microscopy images of texture in 7CB film and Fourier transforms Figure S7: DC voltage-dependent laser beam transmission through 7CB film Video: Pics1.mp4: Pictures of the far-field diffraction pattern behind 5CB film, Pics2.mp4: Pictures of the far-field diffraction pattern behind 5CB film (at a lower laser beam intensity), Movie 1.avi: Time evolution of the light pattern behind AuNPs/5CB film at 4.6 V, Movie 2.avi: Time evolution of the light pattern behind AuNPs/5CB film at 4.9 V, Movie 3.avi: Time evolution of the light pattern behind AuNPs/5CB film at 5 V [92].
