# Supplementary material for: Control of Coherent Light through Microperiodic Director Modulation in Nematic Films under Low-Voltage DC Electric Field
Source: Materials (Basel). 2023 Sep 1;16(17):6014. doi: 10.3390/ma16176014 (PMC10488426; doi:10.3390/ma16176014)
Supplement: Supplementary file 1 [file materials-16-06014-s001.zip › G.Hadjichristov_[Coh.V-shape-5CB]_Supplementary Materials.pdf]

Supplementary

Control of Coherent Light Through Microperiodic Director Modulation in Nematic Films Under Low-Voltage DC Electric Field

Georgi B. Hadjichristov

Laboratory of Optics and Spectroscopy, Georgi Nadjakov Institute of Solid State Physics, Bulgarian Academy of Sciences, 72 Tzarigradsko Chaussee Blvd., 1784 Sofia, Bulgaria

1. Characterization of the coherent light transmittance of planar 5CB films

1.1. Effect from inhomogeneous planar alignment

When the V_{DC} field-induced longitudinal domains (LDs) are less regular due to inhomogeneities in the alignment of the NLC film, the V-shaped transmittance curves are narrower and look like those shown in Figure S1. This is a result of LD disruption due to electrohydrodynamic processes, which at inhomogeneities start at a lower voltage. For such 5CB films whose alignment is less than perfect, the characteristic threshold voltage V_{th} is lower (e.g., 2.6 – 2.7 V) (depending on the laser beam incidence angle) and the curve becomes more symmetric. However, the sharpness of its lower-voltage wing is lost. In any case, the V-shaped minimum remains (if the polarization of the incident laser beam is proper). In particular, Figure S1(b) shows a decrease in the optical contrast ratio T_{max}:T_{min} when a larger area (diameter) of the light passed through the 5CB film was measured. Measured in the same way, the reduction for 5CB films with perfect planar orientation was less.

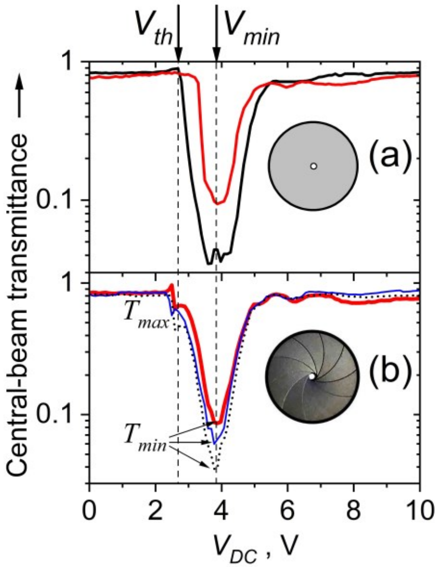

Figure S1. DC voltage-dependent central-beam coherent light transmittance of planar cell with 25 μm-thick 5CB nematic film that had inhomogeneities in the planar alignment. The curves were measured in the far-field zone (85 cm distance from the cell to photodetector with a pinhole) under: (a) normal incidence of He-Ne laser beam; only the center of the central beam separated by a pinhole was detected; two locations on the 5CB film were examined; (b) laser beam incidence angle θ = of 4°; measurements of the central radial portion of the transmitted central beam separated by a pinhole (dotted line), the radial area equal to the FWHM of the radial zone of the transmitted central beam separated by an iris diaphragm (thin line, blue) and the entire central radial zone of the transmitted central beam (bold line, red) separated by an iris diaphragm. In all cases, the laser beam polarization was parallel to the rubbing of the cell plates.

1.2. Temperature behavior

As it is relevant to the practical applicability of the EO control of laser beam intensity by electrically-commanded spatially-periodic director-field modulation in nematic films considered here, the temperature dependence of the V-shaped EO effect observed for the DC voltage-dependent intensity of the central beam of diffraction-transmittance of planar cell with nematic 5CB was inspected. Upon temperature increase in the range from 25 °C to 32 °C (5CB exhibits a stable NLC phase between ~ 24 °C and 34 °C), no significant changes in branch *A* of the curves of V_{DC}-controlled coherent light transmittance were registered within the experimental uncertainty. Also almost identical branch *B* was obtained. As examples, Figure S2 reports two experimental data sets obtained for two examined planar cells with 5CB. Most important for EO applications is the fact that the characteristic voltages V_{th} and V_{min} that define the sharp part to the minimum of the V-shaped characteristic curve of the V_{DC}-dependent coherent light transmittance (i.e., branch *A*), remain the same upon temperature variations in the range 25° C – 32° C.

A detailed inspection of the effect of temperature on the V_{DC}-dependent transmittance curve for the studied 5CB cells revealed some peculiarities, most likely due to temperature-influenced CLS/diffraction arising from the fine LDs, i.e., the narrow parallel stripes. These LDs are field-induced surface flexoelectric deformations in the initially planar-aligned 5CB layers located in a very thin region close to the electrodes of the cell. Within the experimental uncertainty of ± 0.01 V, a small shift to a lower value of V_{min} and a slight narrowing of the transmittance curve with increasing temperature of the 5CB film was found (Figure S2). There was also a small increase of T_{min} value and a corresponding decrease in the dynamic range of the coherent light intensity change (the optical contrast ratio, T_{max} : T_{min}) (more clearly seen in Figure S2b). The trend of an increase of transmitted light intensity with increasing temperature can be explained with the lower optical loss due to reduced light scattering from NLCs at elevated temperature. The latter effect is well known for the nematic state of 5CB [1] and is closely related to the change of refractive index with temperature (also well established for 5CB [2]).

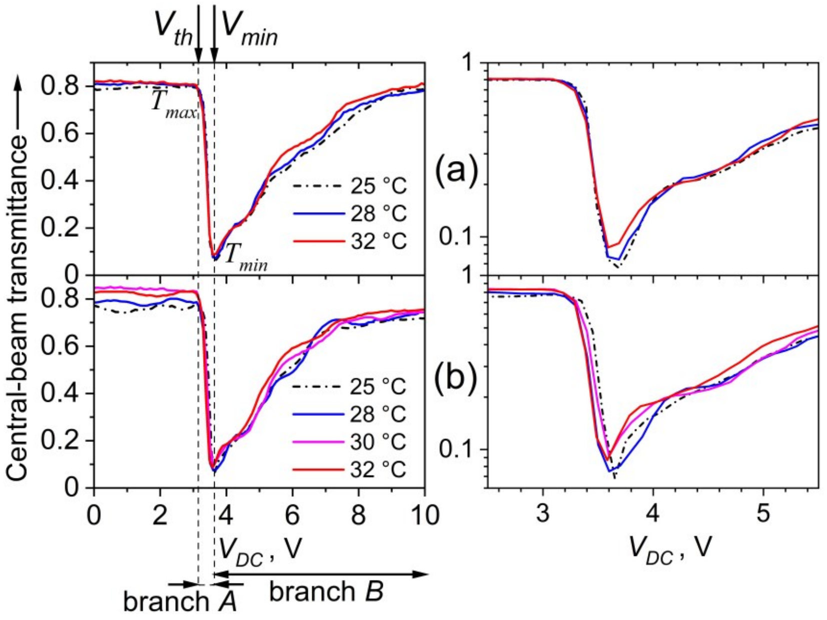

Figure S2. In the left: DC voltage-dependent intensity of the central beam of transmitted-diffracted light behind 25 μm-thick planar 5CB film at various temperature; (a) and (b) show measurements for two 5CB cells under identical experimental conditions. At the bottom are marked the voltage ranges branch *A* and branch *B* of the V_{DC}-dependent transmittance curve. The polarization of the incident He-Ne laser beam was parallel to the rubbing of the 5CB cells. In the right: the same as in the left, but in logarithmic scale for the light intensity and expanded in the voltage range from 2.5 V to 5.5 V.

1.3. Angular behavior

Since the birefringence of the nematics in their interaction with an optical wave depends on the angle of incidence of the light, this angle can influence the EO effect considered here. As an illustration of the variation of the voltage-dependent light intensity of the coherent light transmitted through the studied 5CB planar cell, Figure S3(a,b) presents the measurements for of the central beam of the transmitted light at various angle of incidence of the impinged He-Ne laser beam. As expected, the V_{DC}-dependent coherent light transmittance of the examined planar films was influenced by their orientation toward the Z axis. A deviation from the normal incidence can cause a change in the shape of the central-beam transmission curve. At higher values of the incidence angle, such changes were highly pronounced, especially in the lower-voltage wing of the V-shaped curve, and can distort it. These peculiarities are different from the oscillations in the voltage-dependent coherent light transmittance behaviors typical of NLCs (for example, [3,4]), resulting from voltage-modulated optical anisotropy (birefringence) of the NLCs. This suggests that the coherent light transmittance of the studied 5CB films is affected by accompanying electrically-induced optical processes. It is seen from Figure S3(a,b) that the normal incidence of the laser beam to the nematic film is most favorable for the EO effect considered here.

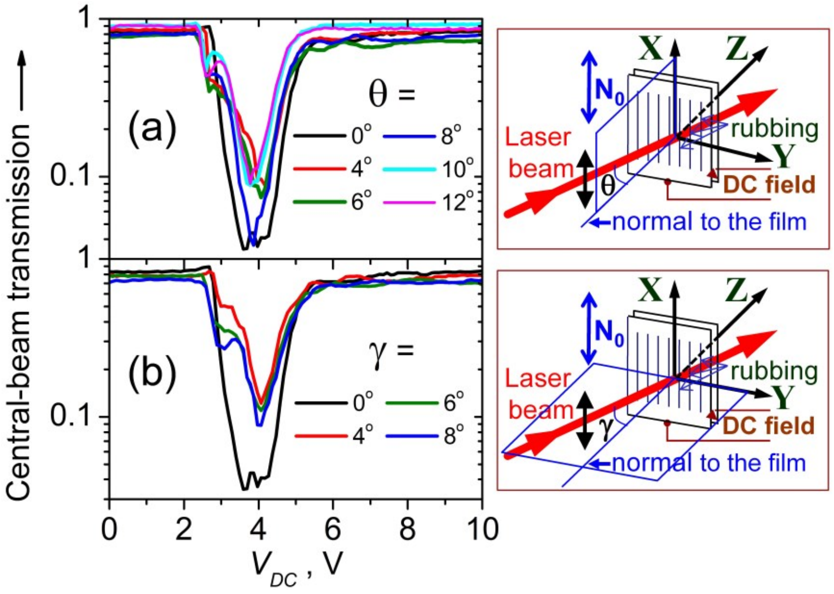

Figure S3. DC voltage-dependent intensity of the central beam of transmitted-diffracted light behind 25 μm-thick planar 5CB cell, at various values of the angle of incidence of the laser beam: polar angle θ in the incidence Z-X plane (a); azimuthal angle γ in the incidence Z-Y plane (b), under the same other experimental condition. In all cases, the location of the illuminated region on the NLC film was strictly kept. The polarization of the incident He-Ne laser beam was along the rubbing of the cell plates. In the right: schematics of the corresponding geometry configuration.

1.4. Effect from the light wavelength

In view of practical application, the EO response of 5CB planar cells was examined upon illumination with coherent light at three different wavelengths (λ), employing three laser sources. Figure S4 compares the V-shaped EO behaviors measured by use of low-divergence c.w. lasers at λ = 632.8 nm (He-Ne laser) and λ = 532 nm (Nd:YVO4 laser), as well as a laser diode emitting at λ = 405 nm, under identical experimental conditions (the same geometry configuration, the same location on the NLC film, the incident light power kept at 1 mW). As seen from Figure S4, a variation of the V-shape of the curves and V_{th} voltage takes place, but importantly, the voltage V_{min} remains nearly the same. The difference in the V-shaped curves recorded at different λ can be attributed to the λ-dependent refractive index of 5CB [5].

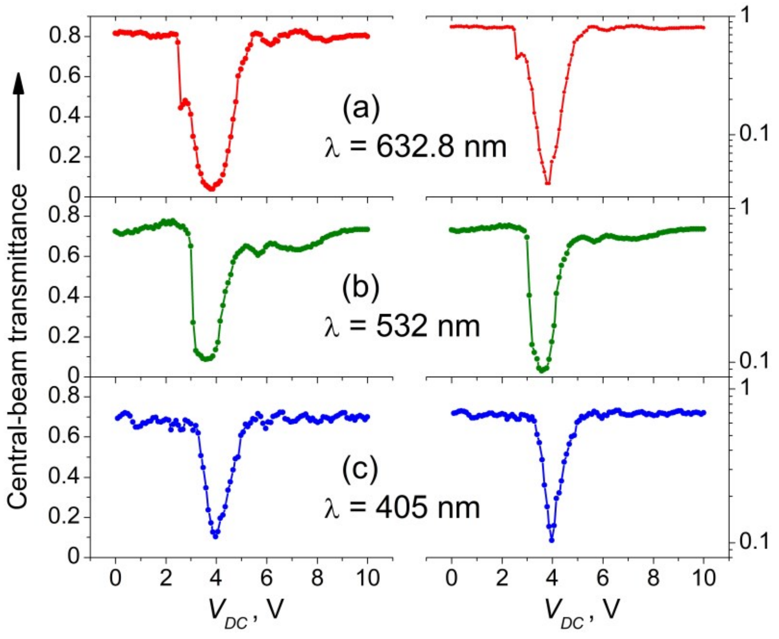

Figure S4. DC voltage-dependent central-beam transmission through a planar cell with 5CB nematic at temperature 28 °C (left – in linear scale; right – in logarithmic scale). The transmittance curves were measured for three wavelengths of the incident laser light, with other experimental conditions being identical. In all cases, the polarization of the incident laser beam was parallel to the rubbing of the cell. Normal incidence of the beam on the 5CB cell.

1.5. Temporal behavior

The voltage-dependent temporal behaviour of the intensity of the light patterns produced by a He-Ne laser beam transmitted through 5CB planar cell was measured by applying a pulse of DC voltage to the cell (Figure S5). This corresponds to a situation of a real-time application of the EO effect considered here. Spatially selected light behind the cell was measured, corresponding to either the central radial area of the coherent light beam, or the light diffracted/scattered due to the coherent optical processes: Fraunhofer diffraction, diffuse CLS and CLSDPs (the latter separated as much as possible from the first-order (+1/–1) Fraunhofer diffraction peaks). As expected, the recorded temporal shapes were quite different and reflect the coherent nature of these scatter/diffraction processes. Some mixing of the individual contributions of these optical processes can not be avoided due to interference, as well as mutual competition between them.

The transmitted central beam whose V_{DC}-dependent EO behaviour is most interesting for practical applictions, exhibits a fairly good EO switching when V_{DC} is close to the value V_{min} corresponding to the minimum of the EO V-curve of transmission (V_{min} = 4 V for the examined 5CB film) (Figure S5a). In this case, following the orientation dynamics of the electrically-driven NLC molecules, the stationary value of the minimized transmitted light intensity is reached within ~ 1 s after some oscillations in the time due to the NLC spatiotemporal instability. As seen from the time response of the 5CB film stressed at $V_{DC} < V_{min}$, the relaxation of the initial deformation of the nematic director in the film may be relatively long – the nematic reaches its steady-state within ~ 7 s (Figure S5b). The oscillations caused by decaying director deformations after the voltage switching-off are an indicator of a complex relaxation process. Stress voltages at a higher V_{DC} level, e.g., above 5V, at which electrohydrodynamic processes in the nematic film start to be induced, result in oscillations during the stressing voltage pulse due to a field-induced deformation and disruption of the wide-formed LDs in the film.

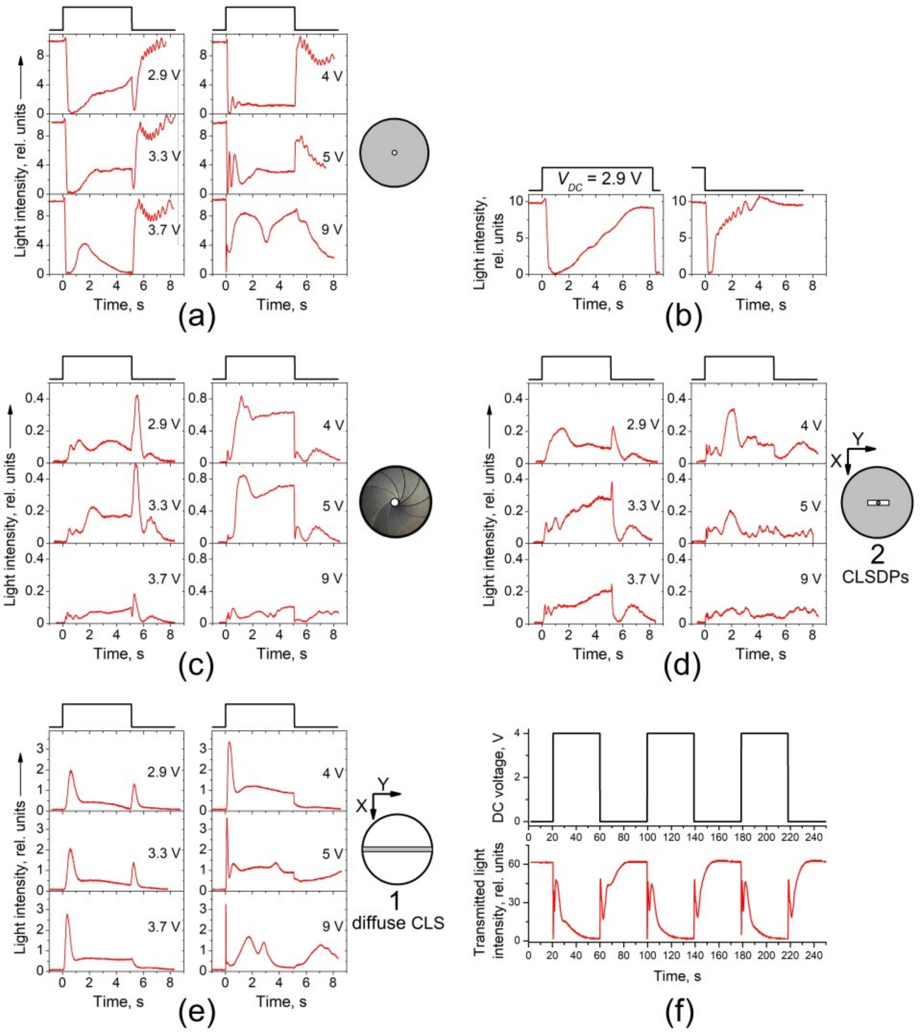

Figure S5. Voltage-dependent temporal behaviour of the intensity of the light patterns produced by a He-Ne laser beam transmitted through 25 μm-thick 5CB planar film by applying various stress DC voltages under identical other experimental conditions. Measurements in the far-field zone (85 cm behind the cell) for separated: (a,b) small radial zone of the central beam; (c) the radial zone of the beam corresponding to the first order of the Fraunhofer diffraction; (d) CLSDPs; (e) diffuse CLS. Time resolution 4 ms. At the top: the stress pulse of DC voltage; the V_{DC} levels are denoted in the graphs. In all cases, the polarization of the laser beam incident normal to the cell was along the rubbing direction of the cell plates. The used spatial filters are sketched in the right of the graphs: the blocked area on the collecting spherical lens is colored in gray. (f) Transient optical response measured for the laser light transmitted through 5CB planar cell under the same experimental conditions as for (a), but at repeating DC pulses with voltage V_{DC} = 4V at the repetition rate of 0.025 Hz. The transmitted light intensity was measured in the near-field region (15 cm behind the cell). The input rectangular DC voltage pulses are plotted in the upper panel.

It should also be pointed out that the time response of the nematic films varies with the laser beam incidence angle (both angles θ and γ, see the previous Section 1.3), as well as the angle φ between the laser beam polarization and the initial director orientation. Note the almost oposite trends that can be observed fro the V_{DC}-dependent EO temporal behaviours of the central-beam transmission (Figure S5a) and those for the spatially localized coherent scattering/diffraction, especially diffuse CLS-relevant light behind the V_{DC}-stressed 5CB cell (Figure S5e). This reciprocity suggests a competition between these field-affected optical processes.

Finally, Figure S5(f) presents the voltage-dependent time response of the examined 5CB planar cell, as measured in the near-field zone. In this case, the recorded temporal trace is a result from the contribution of all the involved scatter/diffractiion coherent proccesses of interaction of the laser light with the nematic 5CB film because they can not be distinguished within the measured radial area of the small-angle cone of transmitted light.

2. Experimental test with nematic 7CB

In general, the optical contrast ratio of the coherent light transmission electro-optically controlled by LDs in NLCs can be improved by use of proper NLC material and varying the system and control parameters, as well as other conditions. A complementary test was performed with identical cells but filled with another cyanobiphenyl NLC, namely heptyl cyanobiphenyl (7CB). Planar-oriented 7CB films were prepared in the same manner as the 5CB films (described in the Experimental part). Like 5CB, 7CB also exhibits a stable nematic phase at room-temperature and positive dielectric anisotropy near to that of 5CB. However, different electric field-induced periodic director pattern formation takes place for both NLCs in identical planar cells.

In the morphology of the 7CB planar films, the stationary narrow-formed rubbing-induced LDs were present, but wide LDs were less pronounced (Figure S6a). Under identical experimental conditions, the measurement of a He-Ne laser beam transmission through planar cells with 7CB indicated a rather weak effect of V_{DC}-induced nematic director modulation (Figure S7a), as compared to that found in 5CB films (discussed in the previous Section). No Fraunhofer diffraction pattern and very weak V_{DC}-induced CLS of the propagating laser beam were registered behind the 7CB cells.

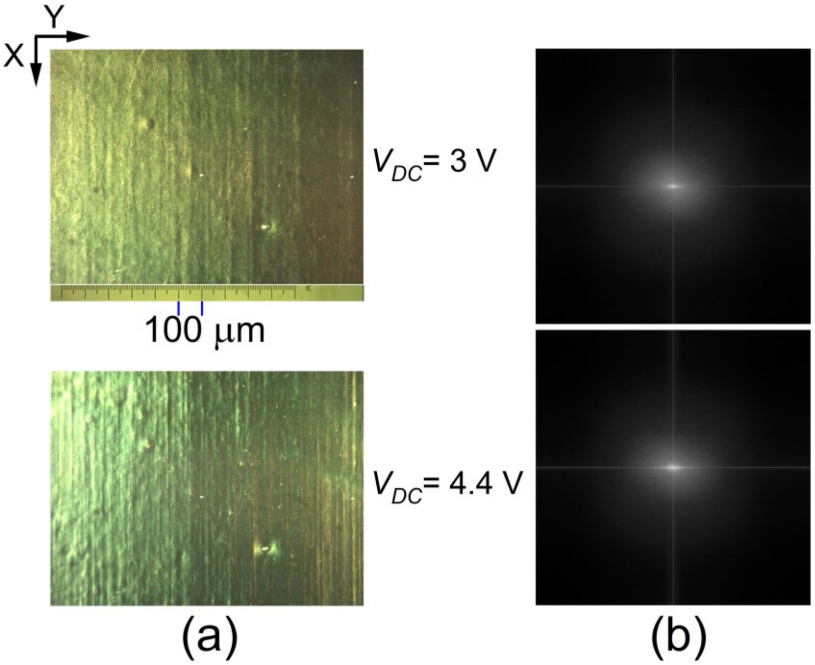

Figure S6. (a) Optical microscopy images of the texture formed in a cell with planar-oriented film (thickness 25 μm) of nematic 7CB, at two values of the applied DC voltage. The micrographs were taken by slightly uncrossed polarizer and analyzer with their axes along *X* and *Y*. The input light polarization was parallel to the rubbing of the cell plates (along the direction *X*); (b) Fourier transforms of the images in (a).

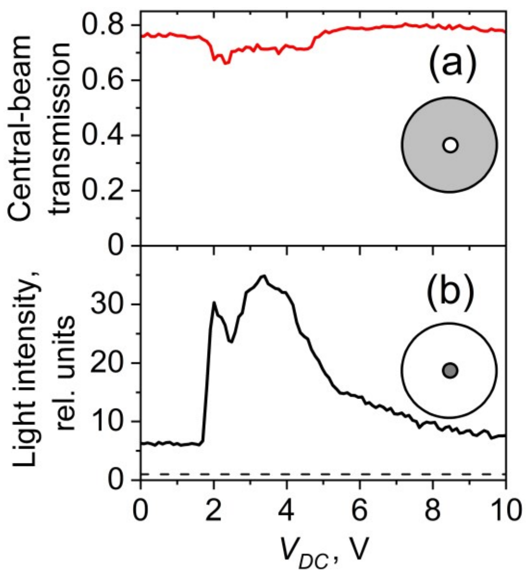

Figure S7. (a) DC voltage-dependent intensity of He-Ne laser beam transmitted through a planar cell with 7CB nematic film (25 µm-thick); (b) V_{DC}-dependent change in the intensity of field-induced CLS from the same cell. The polarization direction of the laser beam was parallel to the rubbing of the cell plates. Both curves were measured under identical experimental conditions except for spatial filtering of the light. The spatial filters are illustrated by inset sketches: iris diaphragm in case (a), and a center-block on the collecting spherical lens in case (b) (the filter-blocked areas are shown in gray). The detection limit (the dark-current background signal) of the apparatus is shown with a dashed line.

Actually, the CLS halo behind the 7CB planar cells at low DC voltages was visible by the eye, but the effect of this diffuse CLS on the central-beam transmittance was negligible. Further, no CLSDPs were present in the voltage range up to 10 V and this was indeed the reason for the very weak reduction effect of the scattering/diffraction from the texture formed in the studied 7CB planar cells. POM showed that the stationary microperiodic narrow-formed rubbing-induced LDs in 7CB planar nematic films (Figure S6a) are less observable than in 5CB films studied in this work. The lack of strong CLSDPs and any localized diffraction patterns agrees with the lack of corresponding features in the Fourier transforms of micrographs of the observed textures. As illustrated in Figure S6(b), only diffusive-concentrated distribution of Fourier amplitudes around zero frequency takes place, in agreement with the diffusive-diffraction halo observed by the experiment. The above experimental evidence highlights the role of the texture formed in the nematic films and shows that the appearance of the specific effect of strong-reduction V-shaped curve for the coherent light transmittance, as by planar 5CB nematic films, is owing to the complex balance between electrohydrodynamic, dielectric and elastic torques, which is reasonable.

References

[1] Strazielle, C.; Coles, H.J. Depolarized light scattering studies of the nematogen pentyl cyanobiphenyl. *J. Phys. (France)* **1979**, *40*, 895–900. https://doi.org/10.1051/jphys:01979004009089500.

[2] Li, J.; Gauzia, S.; Wu, S.T. High temperature-gradient refractive index liquid crystals. *Opt. Express* **2004**, *12*, 2002–2010. https://doi.org/10.1364/OPEX.12.002002.

[3] Blinov, L.M.; Chigrinov, V.G. *Electrooptic Effects in Liquid Crystal Materials*; Springer: New York, USA, 1994; p. 149.

[4] Wu, S.T.; Efron, U.; Hess, L.D. Birefringence measurements of liquid crystals. *Appl. Opt.* **1984**, *23*, 3911–3915. https://doi.org/10.1364/AO.23.003911.

[5] Li, J.; Wen, C.H., Gauza, S.; Lu, R.; Wu, S.T. Refractive indices of liquid crystals for display applications. *J. Disp. Technol.* **2005**, *1*, 51–61. https://doi.org/10.1109/JDT.2005.853357.

3. Pictures

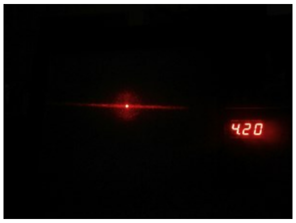

(1) Pics1.mp4 – Pictures of far-field diffraction pattern observed on a black-paper transversal screen behind a planar cell with 5CB film with a thickness of 25 μm. The cell was illuminated with He-Ne laser beam (λ = 632.8 nm) whose polarization was along the direction of the rubbing of the cell plates. The photo series were taken under identical conditions; cell-to-screen distance 85 cm. The values of the DC voltage [V] applied to the cell are indicated.

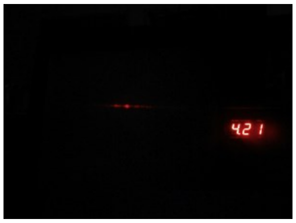

(2) Pics2.mp4 – As for Pics1.mp4, but at a lower intensity of the incident He-Ne laser beam.

4. Videos

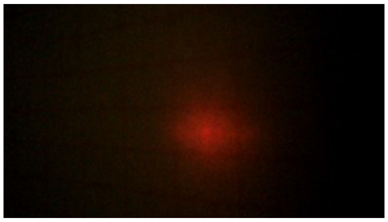

(1) Movie clip 1.avi (30 fps) – Time evolution of the light pattern produced behind AuNPs/5CB planar nematic film illuminated with a beam from He-Ne laser (λ = 632.8 nm) after switching-on a DC voltage of 4.6 V applied to the film. The polarization of the laser beam was along the direction of the rubbing of the cell plates.

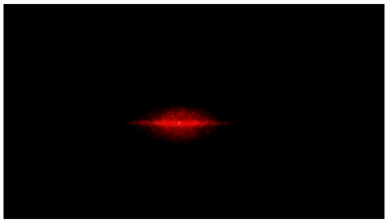

(2) Movie clip 2.avi (30 fps) – Time evolution of the light pattern produced behind AuNPs/5CB planar nematic film illuminated with a beam from He-Ne laser (λ = 632.8 nm) after switching-on a DC voltage of 4.9 V applied to the film. The polarization of the laser beam was along the direction of the rubbing of the cell plates.

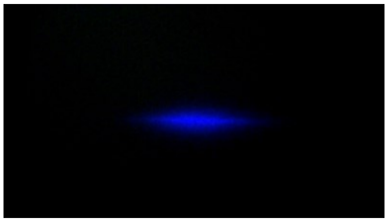

(3) Movie clip 3.avi (30 fps) – Time evolution of the light pattern produced behind AuNPs/5CB planar nematic film illuminated with a beam from a laser diode (λ = 405 nm) after switching-on a DC voltage of 5 V applied to the film. The polarization of the laser beam was along the direction of the rubbing of the cell plates.
